# Environmental instability reduces shock resistance by enriching specialist taxa with distinct two component regulatory systems

**DOI:** 10.1038/s41522-025-00679-w

**Published:** 2025-03-31

**Authors:** Simon Mills, Umer Zeeshan Ijaz, Piet N. L. Lens

**Affiliations:** 1 University of Galway, Galway, Ireland; 2 Water & Environment Research Group, University of Glasgow, Mazumdar-Shaw Advanced Research Centre, Glasgow, UK; 3 Department of Molecular and Clinical Cancer Medicine, University of Liverpool, Liverpool, UK

**Keywords:** Microbial ecology, Applied microbiology

## Abstract

Different microbial communities are impacted disproportionately by environmental disturbances. The degree to which a community can remain unchanged under a disturbance is referred to as resistance^1^. However, the contributing ecological factors, which infer a community’s resistance are unknown. In this study, the impact of historical environmental stability on ecological phenomena and microbial community resistance to shocks was investigated. Three separate methanogenic bioreactor consortia, which were subjected to varying degrees of historical environmental stability, and displayed different levels of resistance to an organic loading rate (OLR) shock were sampled. Their community composition was assessed using high throughput sequencing of 16S rRNA genes and assembly based metagenomics. The effect environmental instability on ecological phenomena such as microbial community assembly, microbial niche breadth and the rare biosphere were assessed in the context of each reactor’s demonstrated resistance to an OLR shock. Additionally, metagenome assembled genomes were analysed for functional effects of prolonged stability/instability. The system which was subjected to more environmental instability experienced more temporal variation in community beta diversity and a proliferation of specialists, with more abundant two component regulatory systems. This community was more susceptible to deterministic community assembly and demonstrated a lower degree of resistance, indicating that microbial communities experiencing longer term environmental instability (e.g. variations in pH or temperature) are less able to resist a large disturbance.

## Introduction

The impact of environmental disturbances on microbial communities has been a central theme of ecological study for decades^1–3^, with implications for global biogeochemical cycling^4^, ecosystem functioning^3^, environmental biotechnology^5,6^ and human health^7^. For example, methanogenic microbial communities play a large role in carbon cycling^8^ and underpin biotechnologies such as anaerobic digestion^9^. These communities are particularly susceptible to disturbances, as they are highly syntrophic and dysbiosis in one of the necessary trophic groups causes system failure^10,11^. The ability of a microbial community to remain unchanged under a disturbance is called resistance, whereas the rate at which microbial composition returns to its original composition after being disturbed is known as resilience^1^. These properties vary among microbial communities, however it is still unclear what causes these variations. One theory is that prior exposure to environmental disturbances increases resistance and resilience^6^. This may be due to changes in underlying ecological properties of the community or the development of new functional traits or adaptations. Ecological properties of a community which may influence resistance and resilience include niche breadth, community assembly and the extent of the rare biosphere.

Niche breadth refers to the extent of a given taxa’s niche, relative to prevailing environmental conditions and is directly influenced by environmental (in) stability^12^. Taxa with wider niches are considered generalists, whereas taxa with narrow niches are considered specialists^13^. It is often stated that generalist taxa will thrive in different environments as they have a wider niche and are not inhibited by variable conditions^13^. In contrast, specialists tend to dominate in stable environments where they can utilise resources more efficiently within a narrow niche^14^. Therefore, historical disturbances are expected to lead to the proliferation of generalist taxa, thus increasing resistance and resilience to further disturbances.

The rare biosphere plays a role in fulfilling niches which are altered due to environmental disturbances by acting as a ‘seed bank’, which supplies new taxa to occupy new or varied niches^15^. In the absence of a disturbance, there are some natural fluctuations in community composition around a stable state of equilibrium, with little opportunities for drastic change in microbial community composition^16^. However, during a disturbance, if total community function is to remain the same, members of the original community must persist (resistance) or be replaced by organisms which can fulfil the same niche, potentially seeded by the rare biosphere^17^. Frequent disturbances can increase the proportion of rare taxa in a community^18^ and may therefore strengthen the likelihood of the rare biosphere fulfilling a new or altered niche space.

The ecological properties of a microbial community which impart resistance may be developed during microbial community assembly or re-assembly after a disturbance. Therefore, frequent disturbances may compound these effects. Microbial community assembly is broadly categorised as stochastic or deterministic^19^. The natural variations in microbiome stability are often stochastic in nature. Deterministic community assembly often refers to external forces which drive community development such as environmental gradients or disturbances, which may modify niches and force the community to change its composition^20,21^.

Functional changes which could occur in response to historical instability may include a more diverse functional capacity or enrichment of stress response mechanisms such as two component regulatory systems (TCRS). Previously TCRSs were enriched during unstable periods of operation in a full scale activated sludge plant^22^. However, the contribution of TCRS to the degree of resistance in an anaerobic microbial community is unknown.

Previously, we reported that historical instability led to the development of a significantly more diverse microbial community in high rate anaerobic digesters^23^. However, this increased diversity did not result in improved resistance, as has been reported in several previous studies^24,25^ and was contrary to our original hypothesis that more diverse communities would have improved resistance. Therefore, in the present study, we further investigated (1) the impact of environmental instability on ecological properties of these communities over time; and (2) The ecological and functional characteristics of a community with decreased resistance. Three laboratory microcosms of methanogenic microbial consortia were subjected to varying degrees of environmental stability before receiving a massive influx of excess carbon, which typically results in acidosis and community dysbiosis^23^. In depth ecological analysis of the microbial communities prevailing in each of these microcosms revealed that historical environmental instability led to a community with inflated numbers of specialist taxa and reduced resistance to an organic shock load.

## Methods

### Experimental design

**Fig. 1:**
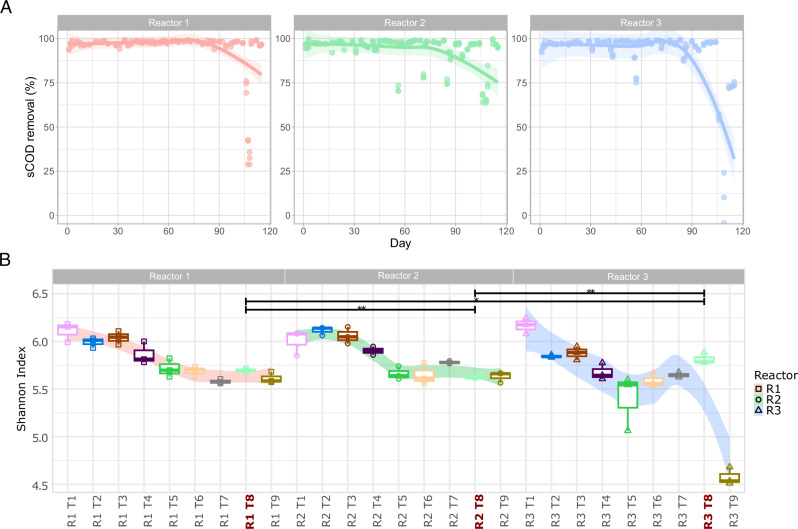
Overview. **A** sCOD removal efficiency (%) throughout the course of the trial in reactor 1 (R1), reactor 2 (R2) and reactor 3 (R3). Timing of applied disturbances and the final shock are indicated by coloured stars. **B** Alpha Diversity (Shannon Entropy) at each timepoint (T1–T9) in the groups R1, R2 and R3. T8 in all reactors is bolded and in red for clarity. Lines of significance depict significant differences in alpha diversity between the T8 samples, prior to the OLR shock as follows: *(*p* < 0.05), **(*p* < 0.01), or ***(*p* < 0.001) based on ANOVA). Only T8 was included in significance testing.

Granular sludge was sampled from three expanded granular sludge bed (EGSB) bioreactors operated at 37 °C which were subjected to varying degrees of environmental stability^23^. Associated physiochemical parameters were reported previously^23^ and are summarised in Fig. 1. The bioreactors were supplied with a synthetic dairy wastewater (3.5 g COD/l) consisting of skimmed milk powder, trace elements and macronutrients at an 18 hr HRT. The influent was buffered with 1 g/l sodium bicarbonate. Reactor 1 (R1) was maintained stable for the entire period of operation. After a period of ~40 days reactor 2 (R2) and reactor 3 (R3) were subjected to various small disturbances (D1–D4) through variations in influent composition (Fig. 1). R2 was subjected to repeated disturbances in the form of excess carbon influxes by increasing the concentration of the influent. R3 was subjected to a range of disturbances which are common in methanogenic bioreactors including ammonium and sulphide toxicity as well as a temperature and pH drop (Fig. 1). Subsequently, all three bioreactors were subjected to a massive carbon influx by increasing the chemical oxygen demand (COD) concentration of the feed 8-fold to 28 g COD/l.

### 16S rRNA gene amplicon sequencing

Samples for 16S rRNA gene amplicon sequencing were taken from each reactor at 9 timepoints (T1–T9) throughout the experiment, before and after a disturbance was applied (Fig. 1). Cell lysis was performed by beat beating with 1% cetyltrimethylammonium bromide (CTAB) and total genomic DNA was extracted using a phenol:chloroform based extraction protocol^26^. DNA was quantified using a qubit fluorometer (Invitrogen, Carlsbad, CA, USA) and checked for quality by visualisation on agarose gel electrophoresis and purity on a NanoDrop spectrophotometer (ThermoScientific). PCR amplification of partial 16S rRNA genes was carried out using the 515F 806R primer pair^27^. PCR cycles were as follows: initial denaturation was performed for 3 min at 95 °C, followed by 25 cycles of denaturation at 95 °C for 30 s, annealing at 55 °C for 30 s, and extension at 72 °C for 30 s. Amplicon sequencing was done on the Illumina MiSeq platform by the Foundation for the Promotion of Health and Biomedical Research of Valencia Region (FISABIO) (Valencia, Spain). Metagenomic sequencing was carried out by Novogene on a NovaSeq 6000 instrument using V1.5 reagents. Statistical analysis was performed using R software and the details are provided in the Supplementary Material. Raw amplicon sequencing data used in this study has been deposited in the National Center for Biotechnology Information (NCBI) Sequence Read Archive (SRA) under the BioProject accession number PRJNA1030189. Metadata used in the analysis of sequencing data has also been provided as Supplementary Files 1 (amplicons) and 2 (metagenomes).

### Metagenomic analysis

A total of 18 metagenomics samples were processed. These samples were taken from all three reactors at timepoint 1(T1) prior to any disturbances and timepoint 8 (T8) prior to the large OLR shock (Fig. 1). Reads were quality trimmed using Sickle v1.200^28^ at an average Phred quality of <20. Reads which were >50 bp were retained after trimming for downstream processing. This gave a total of 641,256,041 reads from all samples. All forward and reverse reads were then collated together together for co-assembly using megahit with the parameters—k-list 27,47,67,87—kmin-1pass -m 0.95—min-contig-len 1000^29^ his gave us a total of 653,274 contigs, a total of 213,972,1678 base pairs (bp), maximum of 654,587 bp, average length of 3275 bp and an N50 score of 4774 bp.

The MetaWRAP pipeline^30^ was used to bin the contigs with three different binning algorithms: metabat2 (610 bins), maxbin2 (520 bins) and CONCOCT (381 bins). Checkm^31^ was applied to the completion and level of contamination of each bin. The bins from the three binners were consolidated together within the MetaWRAP framework, only retaining bins with $\ge$50% completion and $\le$10% contamination to give a final set of 380 bins or metagenomic assembled genomes (MAGs). We applied CheckM^31^ on these bins to assess their completion and contamination. Within MetaWRAP framework, the bins from the three binners were consolidated together, retaining bins with >50% completion and <10% contamination to give a final set of 380 bins (MAGs). We obtained a mean genome completion of 78.68% and a mean contamination of 2.29% for bins. To deduce the phylogeny of the Metagenome-Assembled Genomes (MAGs), we employed GToTree^32^. The software offers various Single Copy Genes (SCGs) sets based on the resolution of domains and the taxonomic rank of interest. Specifically, we utilised two SCG sets: a 25-gene set for Bacteria and Archaea (resulting in the phylogeny recovery for 275 MAGs). Finally from 380 bins, for downstream statistical analysis, we have used bins with >75% completion, i.e. 219 bins. The METABOLIC^33^ pipeline was used to assign functions to each bin using GTDB-TK^34^. METABOLIC was also used for the annotation of proteins using KEGG^35^. The obtained sample read coverages per bin ${C}_{i,j}$ was then multiplied with feature coverages (returned from METABOLIC) per bin ${F}_{j,k}$ to obtain feature coverages per sample ${n}_{i,k}$ as a matrix product ${n}_{i,k}=\sum _{j}{C}_{i,j}{F}_{j,k}$. Only bins with a genome completeness of $\ge$75% and genome contamination $\le$ 5% were used, resulting in a final dataset of *n* = 18 × *p* = 380 bins. Abundance tables for the following databases were then also generated: KEGG Modules (*n* = 18 samples × *p* = 764 features, i.e. peptidases and inhibitors) and KEGG Submodules (*n* = 18 samples × *p* = 2235 features). All datatables and code used in the analysis of amplicons and metagenomes has been made available on Zenodo^36^ (10.5281/zenodo.14845121).

## Results

### Physiochemical data

Physiochemical, bioreactor performance data and basic measures of alpha and beta diversity were reported in detail previously^23^ and are summarised in Fig. 1. After the application of different disturbance regimes (Table 1) were applied to each reactor, their resistance was assessed by applying a large OLR shock and measuring their sCOD removal efficiency. The mean removal efficiencies of R1, R2 and R3 during the stable period before the shock load (days 99–104) were 97.9%, 97.0% and 97.9% respectively, prior to application of the OLR shock on day 105. In response to the shock the sCOD removal efficiency of R1 decreased to 73.4% and R2 decreased to 66.9%, before both recovered after 7 days. After the shock the pH of R1 and R2 decreased to ~6 (Supplementary Fig. 1). The sCOD removal efficiency of R3 was impacted the most, decreasing to 55.5% and not recovering for the duration of the trial (70–75%) sCOD removal, causing a decrease in pH to nearly 5 (Supplementary Fig. 1). Therefore, R1 demonstrated the most resistance to the shock in terms of performance (soluble COD removal), R2 performed similarly, although slightly worse than R1, whereas R3 performed the worst overall and did not recover to previous levels of performance by the end of the experiment (Fig. 1)^23^. Sludge volumes in each reactor fluctuated throughout the trial, especially in R2 and R3 (Supplementary Fig. 3), where flocculent sludge growth was observed after each disturbance. Alpha diversity in R3 was significantly (*p* < 0.01) higher in R3 than R1 or R2, prior to the application of the shock load (Fig. 1).

**Table 1:** Disturbance regimes applied to R1, R2 and R3

Day	Reactor	Event/Sampling	Reduction in sCOD removal (%)
Day 41		T1- Amplicons, Metagenomes	
Day 41	R1	None	N/A
	R2	Ammonia disturbance (3 g/l for 24 h)	4.3
	R3	2-fold OLR increase	1.7
Day 54		T2 - Amplicons	
Day 55	R1	None	N/A
	R2	4-fold OLR increase	4.9
	R3	Temperature drop (15 °C for 24 h)	8.7
Day 64		T3 - Amplicons	
Day 69		T4 - Amplicons	
Day 70	R1	None	N/A
	R2	4-fold OLR increase	5
	R3	pH decreased (pH 3.8 for 24 h)	4.1
Day 78		T5 - Amplicons	
Day 84		T6 - Amplicons	
Day 84	R1	None	N/A
	R2	4-fold OLR increase	9.1
	R3	Sulphide addition (600 mg/l for 24 hr)	9.3
Day 92		T7 - Amplicons	
Day 105		T8 - Amplicons, Metagenomes	
Day 105	R1	8-fold OLR increase	24
	R2		30.4
	R3		42.6
Day 113		T9 - Amplicons	

In addition, each reactor was sampled at 9 different timepoints throughout the trial (T1–T9) and alpha diversity analysis indicated that the variable environmental conditions of R3 led to significantly (*p* < 0.01) higher Shannon diversity at T8, right before the applied shock^23^ (Fig. 1).

### Microbial community temporal dynamics

**Fig. 2:**
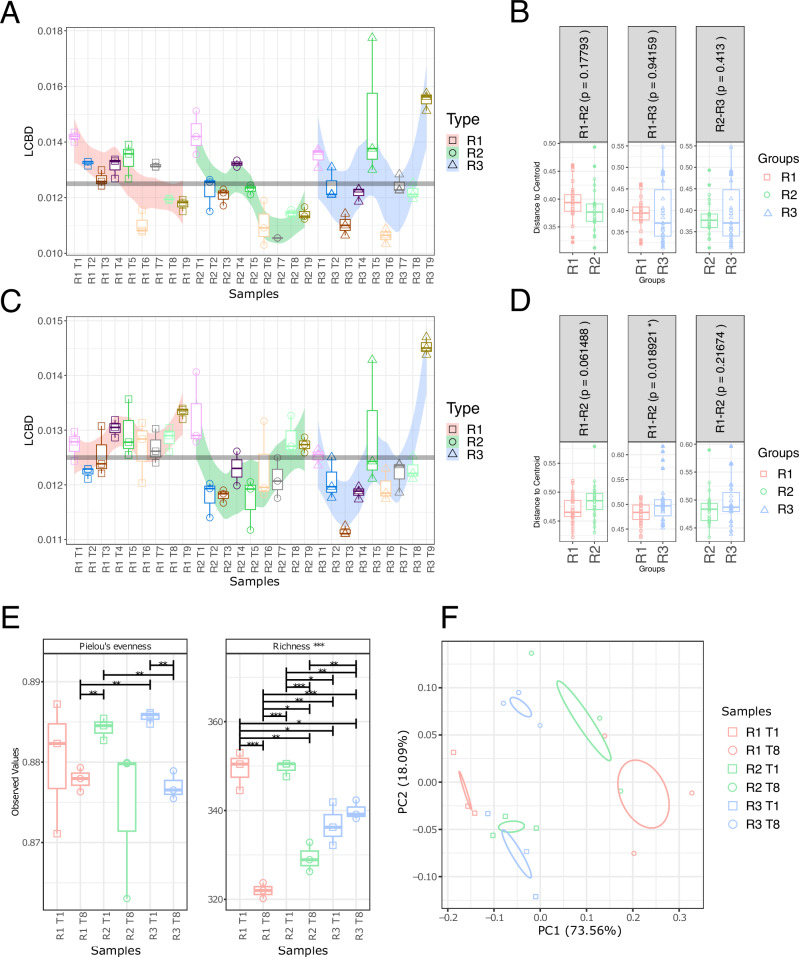
Beta diversity. **A** Local contribution to beta diversity (LCBD) using the Bray-Curtis distance metric for reactor 1 (R1), reactor 2 (R2) and reactor 3 (R3) where the horizontal grey bar indicates mean beta diversity across all samples. **B** Pairwise comparisons of beta dispersion among samples from R1, R2 and R3 using the Bray-Curtis distance metric (statistical significance is denoted as follows: * (*p* < 0.05), ** (*p* < 0.01) or *** (*p* < 0.001) based on ANOVA) for pairwise comparisons. **C** LCBD diversity using the Unifrac distance metric, for R1, R2 and R3. **D** Pairwise comparisons of beta dispersion among samples from R1, R2 and R3 using the Bray-Curtis distance metric. **E** Alpha diversity indices Pielous Evenness and Richness for KEGG modules detected in assembled MAGs (Lines of significance depict significant differences as follows: * (*p* < 0.05), ** (*p* < 0.01) or *** (*p* < 0.001) based on ANOVA for all reactors and timepoints. **F** Principal Co-ordinate Analysis of detected KEGG module abundance with the bray-curtis distance metric in assembled MAGs, where samples were grouped by reactor (R1, R2 and R3). In the boxplots, centre value lines indicate the median, boxes indicate the lower/upper quartiles (25%/75%) and lines extending parallel from the boxes (whiskers) show the variability outside the upper and lower quartiles. Ellipses were drawn using 95% confidence intervals based on standard deviation. Sampling timepoints are denoted as T1–T8 in all panels. Beta dispersion groups R1, R2 and R3 include all timepoints throughout the trial in each reactor.

The local contribution of each sample to the mean beta diversity (LCBD), across all samples was calculated to identify which reactor had more variation in microbial community composition over time (Fig. 2). Beta diversity varied more in R3, when measured in terms of ASV abundance (Bray-Curtis) and phylogeny (unifrac) (Fig. 2), particularly at T9. Beta dispersion analysis was applied to identify and compare the degree of inter-sample variability within groups (reactors). Significant (*p* < 0.05) differences in phylogenetic (unifrac) beta dispersion were identified between R3 and R1, indicating that the phylogenetic composition of R3 was more variable over time than that of R1.

**Fig. 3:**
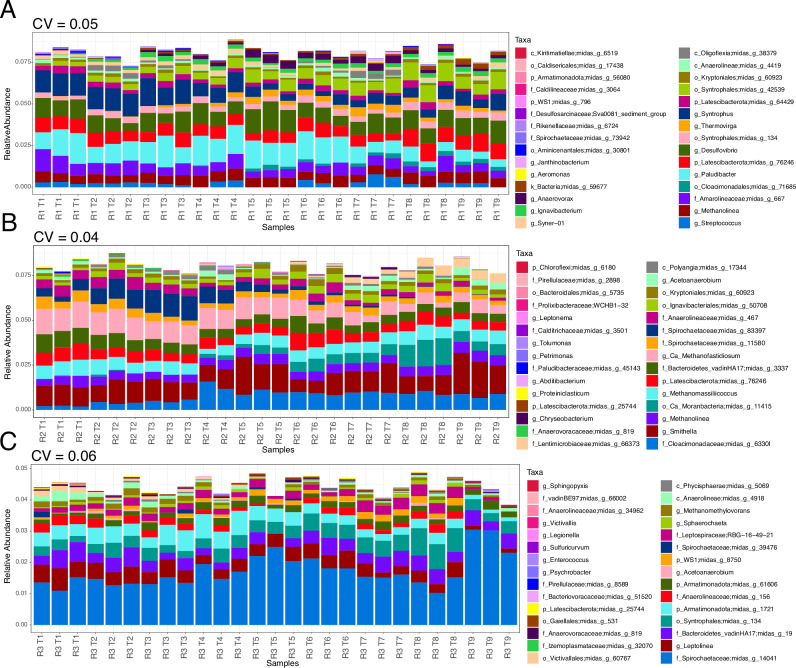
Microbiome stability. Stable component of each reactor’s microbial community returned after running the ensemble quotient analysis (EQO) algorithm in uniform phenotypic variable mode showing the relative abundance profiles of stable taxa in reactor 1 (R1) (**A**), reactor 2 (R2) (**B**) and reactor 3 (R3) (**C**) with *Coefficient of Variation* (CV) values given on the top of the plot. The lower CV value signifies higher stability. Sampling timepoints are denoted as T1–T8 in all panels. Taxa are represented at the highest meaningful taxonomic level, with the accompanying midas field guide genus code, where genus could not be assigned. Taxonomic levels are denoted as follows; p Phylum, c Class, o Order, f Family, g Genus.

EQO analysis^37^ was used to identify a minimal set of microbes (limited to 30), which remained stable across sampling timepoints, with a minimum coefficient of variation. These taxa remained stable in composition in response to the applied disturbance regimes (Fig. 3). The coefficient of variation for the stable community of R3 was higher (CV: 0.06) than that of R1 (CV: 05) and R2 (CV: 04), indicating that although this community was considered stable in its own right, it was more variable than the communities present in R1 or R2. The stable communities of each reactor also differed in composition (Fig. 3). The stable community of R1 was dominated by *Streptococcus* sp., *Methanolinea* sp., *Paludibacter* sp. and *Desulfovibrio* sp., as well as members of the Anaerolinaceae family (Midas_g_667) and the order Cloacimonadales (Midas_g_71685). The stable community of R2 was dominated by *Smithella* sp., *Methanolinea* sp., *Methanomassiliicoccus* sp., as well as members of the family Cloacimonadaeae (Midas_g_63301). The stable community of R3 was heavily dominated by a member of the Spirochaetaceae family (Midas_g_14041), particularly at T9 after the shock load, with lower abundances of *Leptolinea* sp, genera from the Bacteroidetes_vadinHA17 family (Midas_g_19) and the order Syntrophales (Midas_g_134).

**Fig. 4:**
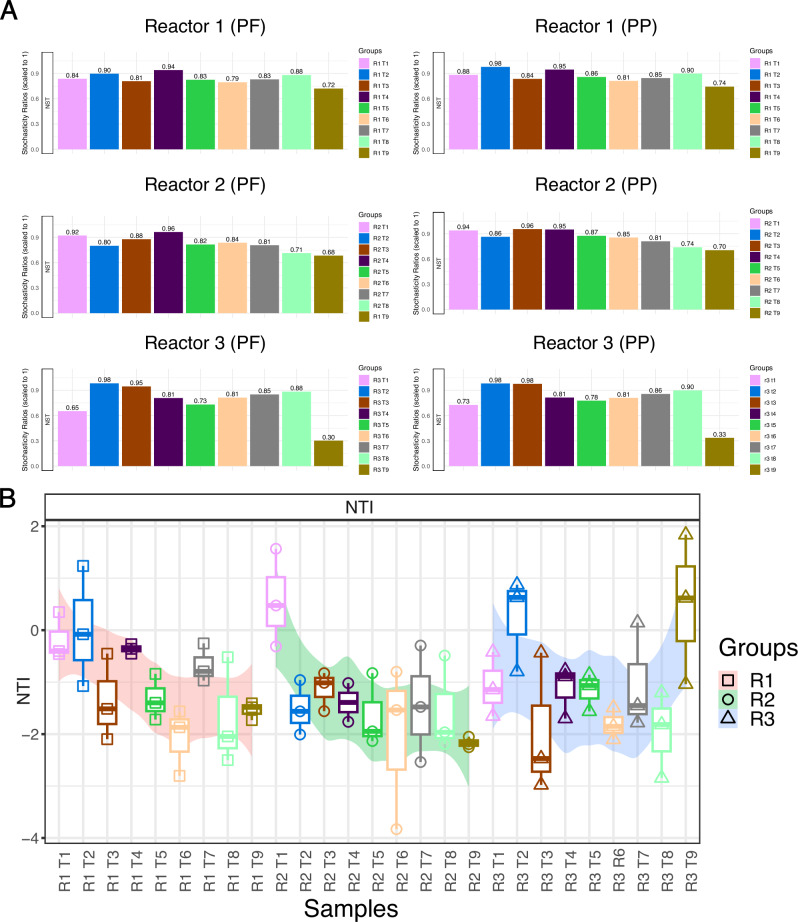
Microbial community assembly. **A** Normalised stochasticity ratio (NST) using Ružička (abundance based) distance metric and Taxa-Richness constraints of proportional-fixed (PF) and proportional-proportional (PP) abundances for Reactors 1–3. **B** NTI values for all samples, sorted by reactor and timepoint. Reactors are denoted as R1–R3, sampling timepoints are denoted as T1–T8 in all panels.

The relative contributions of stochastic and deterministic influences on community assembly were assessed using two methods; the nearest taxon index (NTI) and normalised stochasticity ratio (NST)^38^, both of which revealed similar patterns. Both NTI and NST are ‘null modelling’ procedures, where artificial, randomised communities are randomly generated in high numbers (e.g. 1000) and compared to the the original community. The deviation of the real community from this randomised null model in terms of a given metric such as beta diversity or phylogenetic relatedness then leads to inferences about ecological principals. The difference between NTI and NST is in the choice of the distance metric uses. NTI uses nearest taxon distance (calculated on the phylogenetic tree as a proxy for local clustering) whilst NST can use any beta diversity distances (both incidence and abundance) as long as the distance can be normalised between the values of 0 and 1. There are subtle differences in mathematical derivation of both measures (see Supplementary Material for further info), with NTI relying on ‘statistical effect size’, whilst NST uses a bespoke ‘selection strength’ metric. However, both methods give a threshold on the bases of which one can decide the influence of the environment and presence of stochasticity. A positive NTI value indicates clustering in the phylogenetic tree, which can imply deterministic influences, whereas a negative value indicates phylogenetic dispersion, suggesting that stochastic processes dominate. For NST community assembly is considered entirely deterministic when NST is 0% and entirely stochastic when NST is 100%. A 50% stochasticity ratio separates deterministic (<50%) and stochastic (>50%) assembly processes^38^. Community assembly in all 3 reactors was largely stochastic in nature (i.e. NST > 0.5) and remained fairly stable in R1 and R2 (Fig. 4). However, there was a slightly different temporal pattern in R3, which showed a slight curve over the course of the trial, indicating that stochasticity remained high, but decreased slightly until T5, before increasing again to T8 (Fig.4). The final timepoint in R3 however, experienced a drastic increase in deterministic influences, as evidenced by both NST (<0.5) and NTI (>2) values.

### Ecological and functional properties

In some instances, rare taxa are thought to contribute to maintaining stable community functioning by replacing abundant taxa which have been inhibited by an environmental disturbance^15^. These organisms which take advantage of the change in environmental conditions are known as conditionally rare taxa. In the present study, we assessed the fluctuations of rare taxa in order to determine if the presence of conditionally rare taxa contributed to the greater functional resistance observed in R1 and R2.

The relative contribution of abundant, conditionally rare, persistently rare and other rare taxa was calculated according to the methods outlined by Yang et al.^39^. Conditionally rare taxa exhibit a maximum relative abundance at least 100 times higher than its minimum value. Persistently rare taxa never exhibited a maximum relative abundance 5 times greater than its minimum. Other rare taxa exhibited a maximum relative abundance between 5 and 100 times greater than it’s minimum^39^.

**Fig. 5:**
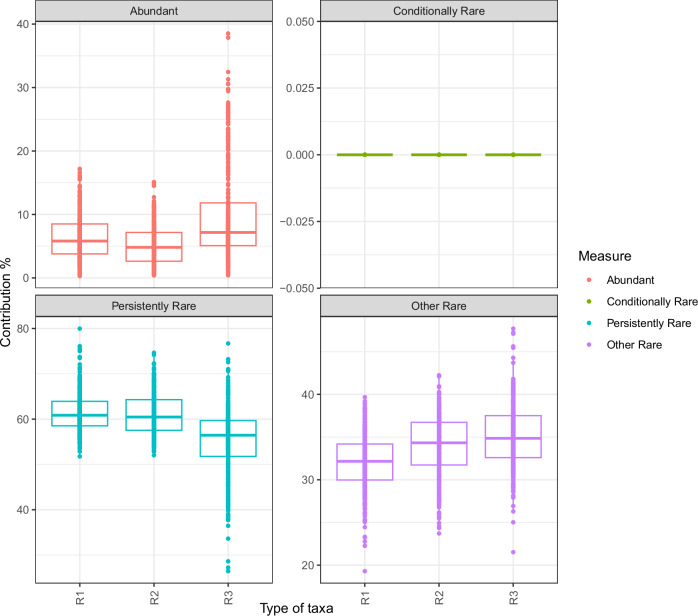
Rare taxa. Boxplots depicting abundant (taxa which exhibit average relative abundance above 1% across all samples), conditionally rare (taxa which exhibit a maximum relative abundance at least 100 times higher than its minimum value), persistently rare (as taxa which never exhibited a maximum relative abundance 5 times greater than its minimum) and other rare (taxa which exhibited a maximum relative abundance between 5 and 100 times greater than its minimum) taxa in Reactors 1-3 (R1–R3). In the boxplots, centre value lines indicate the median, boxes indicate the lower/upper quartiles (25%/75%) and lines extending parallel from the boxes (whiskers) show the variability outside the upper and lower quartiles. Groups R1 (reactor 1), R2 (reactor 2) and R3 (reactor 3) include all timepoints throughout the trial in each reactor.

No conditionally rare taxa were identified in any of the reactors investigated (Fig. 5). However, some trends were apparent within the persistently rare taxa and other rare taxa. The contribution of persistently rare taxa decreased from R1 to R3, meaning that R3 had the least persistently rare taxa and responded worst to the large OLR shock. The inverse was observed for other rare taxa (Fig. 5). This indicates that there were no large fluctuations in taxa abundance, resulting in conditionally rare taxa (i.e. maximum relative abundance at least 100 times higher than its minimum value). However, R3 did have more taxonomic fluctuations resulting in the presence of other rare taxa (i.e. maximum relative abundance between 5 and 100 times greater than its minimum) (Fig. 5).

**Fig. 6:**
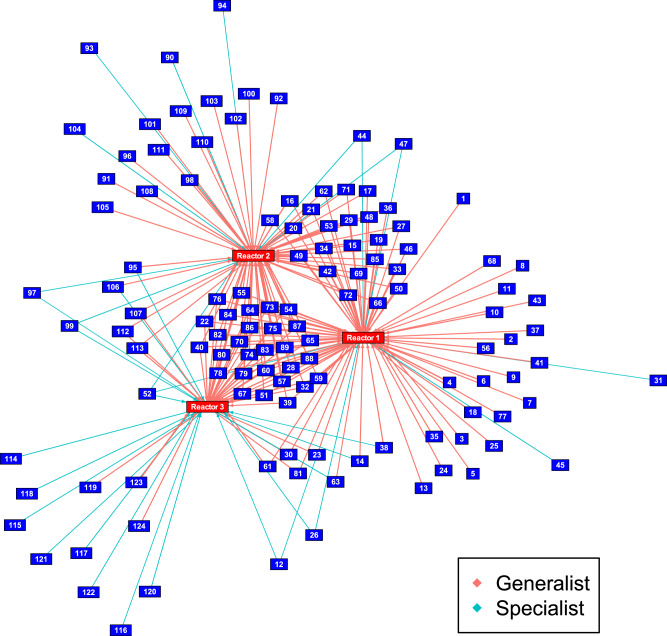
Microbial generalists and specialists. Network of relationships recovered after applying Levin’s B_N_ to find generalists and specialists. Groupings in this case include only timepoints T1–T8 (i.e. omitting T9) in each reactor in order to determine the distribution of generalists and specialists prior to the application of the OLR shock. Numbers indicate individual taxa which were identified as generalists or specialists, these taxa can be found in Supplementary Table 1. Connecting lines indicate whether a taxa was a generalist or a specialist and which reactor they were associated with.

Different microbial life strategies can be more suited to unstable environmental conditions and allow improved functional resistance to environmental change^13^. For example, generalist taxa, with broader niches can thrive under a variety of conditions in comparison to specialists, which thrive in a narrow environmental niche^13,40^. In order to determine if the applied disturbance regimes led to a change in the proportion of specialsts to generalists, the MicroNiche^41^ package was used. For this analysis each reactor was considered to be a separate environment and only timepoints 1–8 (prior to the OLR shock) were considered. In total, 124 genera were identified which fit the criteria for having a generalist or a specialist lifestyle across all three reactors. R1 contained 82 generalists and 7 specialists, R2 contained 71 generalists and 9 specialists (Fig. 6). R3 however, contained only 39 generalists but had 18 specialists (Fig. 6). The overlap between these 124 taxa was also considered to identify taxa which coincided with each other. Supplementary Figs. 4–6 show that there was much less overlap amongst the taxa in R3 due to the presence of more specialists. A full list of specialist and generalist taxa and their classification in relation to each reactor is provided as Supplementary Table 1.

**Fig. 7:**
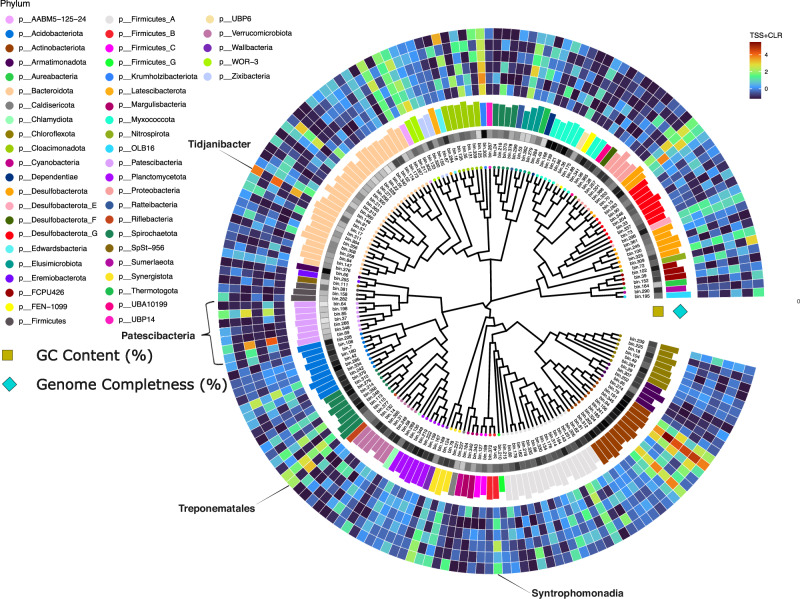
Phylogenetic tree. Krona Plot of metagenomic bins. Bin statistics and phylogeny can be found in Supplemental File 4. Samples are arranged using single copy genes for bacteria and archaea. Abundance of each bin is indicated as a heatmap. Genome completeness (%) is indicated as a bar plot. GC content (%) is indicated as heatmap.

After the initial recovery of 380 MAGs with completeness >50% and contamination <10% we only used MAGs with a completeness greater than 75% and contamination of less than 5% for downstream analysis (*n* = 219), the most abundant of which included Methanothrix, Brevilactibacter, Brooklawnia and Tidjanibacter species (Supplementary Fig. 2). Phylogenetic marker genes for 183 of these bins were identified and aligned using phylogenetic marker genes for bacteria and archaea to determine the phylogenetic distribution of MAGs. Several bins were more abundant in all three reactors at T8 including bin 57 assigned to the genus *Tidjanibacter* and bins 115 and 46 assigned to the orders Treponematales and Syntrophomonadia (Fig. 7). Three bins (bin. 69, bin.198 and bin.348) within the Patescibacteria phylum (also known as candidate phyla radiation) were also more abundant at T8 than at T1, including one which was included in the 20 most abundant taxa (Fig. 7), assigned to order level as Moranbacteriales. Both of these bins became especially abundant in R1 and R2 at T8 but were not as dominant in R3 (Fig. 7).

**Fig. 8:**
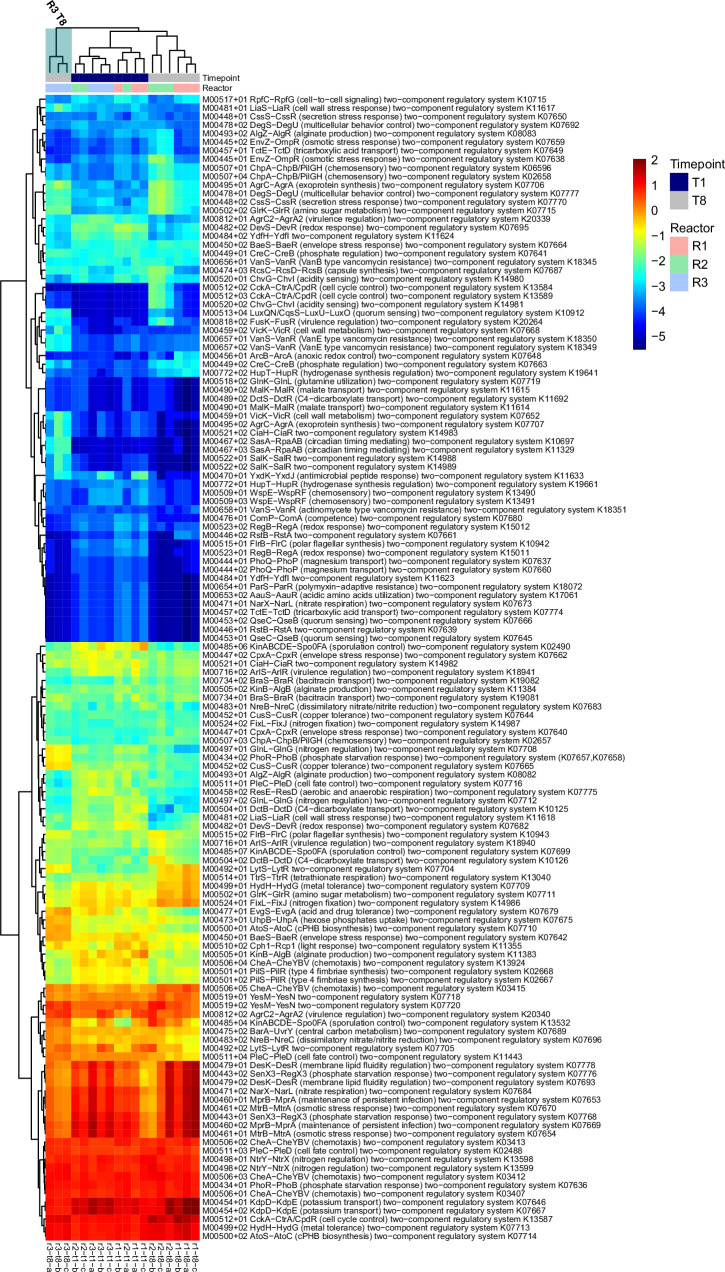
Two component regulatory systems. Abundance of two component regulatory systems across all MAGs recovered from all metagenomic samples (Reactors 1–3 Timepoints 1 and 8). Individual samples are indicated on the x axis with replicates denoted a–c. Samples are ordered on the *x* axis by hierarchical clustering. R3 T8 is highlighted using a blue box. Phylogenies and bin statistics are provided as Supplementary File 4.

The richness of KEGG modules detected decreased significantly over time in R1 (*p* < 0.001) and R2 (*p* < 0.001) but there was no significant change in R3 (Fig. 2). However, evenness of KEGG modules in R3 was significantly (*p* < 0.01) lower at T8, indicating that the functional profile of the community was dominated by fewer KEGG modules. A similar clustering pattern was observed when detected among KEGG modules when principal coordinate analysis (PCoA) was applied, where samples from each timepoint were distinct (Fig. 2). The abundance of different KEGG sub-modules in MAGs, across different module categories was determined (Supplementary File 3) and analysed to identify differing functional potential across samples. The abundance of sub-modules associated with TCRS was of particular interest, as these are known to act as a response mechanism for environmental stimuli. Therefore, the presence/absence of TCRS may infer a degree of resistance to environmental disturbances. Indeed, the abundance of TCRSs was variable among samples, with R3 timepoint 8 (R3T8) forming a distinct cluster (Fig. 8). 82 TCRS were more abundant in R3T8 than in R1 timepoint 8 (R1T8). Several distinct clusters were key in differentiating R3T8 from the other samples, particularly R1T8. TCRS within these clusters which were more abundant at T8 included LuxQN/CqsS−LuxU−LuxO (quorum sensing), VicK−VicR (cell wall metabolism), AtoS−AtoC (cPHB biosynthesis) and CiaH−CiaR. TCRS which were more abundant in R1T8 included ArcB−ArcA (anoxic redox control) and HupT−HupR (hydrogenase synthesis regulation). TCRS which were more abundant in R1T8 than R3T8 included ArcB−ArcA (anoxic redox control) and HupT−HupR (hydrogenase synthesis regulation) (Fig. 8).

## Discussion

This study found that unstable environmental conditions (in R3) resulted in the development of a microbial community rich in specialist taxa (Fig. 6), with distinct TCRS (Fig. 8) and less resistance to a shock load. After application of the shock, there was a large shift from stochastic community assembly to deterministic community assembly (Fig. 4) and ultimately, failure of the system. In contrast, a stable environment (in R1) led to the proliferation of generalist taxa (Fig. 6) and a community which was more resistant and less impacted by deterministic influences after a shock.

The failure of R3 was surprising as the microbial community at T8 prior to the application of the final shock load had a significantly (*p* < 0.01) higher alpha diversity (Fig. 1) than R1 or R2. This is contrary to a lot of research which demonstrates that increased diversity leads to increased stability, resistance and functioning in other anaerobic bioreactors treating different types of wastewaters^24,25^, but also other environments including the human gut^42^ and soils^43–45^, thus indicating that it is not diversity itself which imparts resistance, but some underlying ecological or functional property of the community.

The communities of R1 and R2 remained more compositionally stable than R3 throughout the course of the trial and community beta diversity fluctuated less in R1 and R2 than in R3 (Fig. 2), indicating that the unstable environment of R3 led to more changes in microbial community composition. Environmental fluctuations have been suggested to influence diversity by creating new niches faster than species go extinct, leading to increased overall alpha diversity^46^. However, R2, which received OLR disturbances did not exhibit the same degree of compositional fluctuations or the increase in alpha diversity. This may be because the influent was only varied in concentration rather than composition, therefore making it less likely that new niches developed in this reactor. The environmental variations and resultant community fluctuations in R3 led to significantly higher alpha diversity at T8, before the shock load. However, this did not result in a more resistant community, in terms of function or composition. Generally, higher diversity is thought to give more functional stability and functional resistance to a community^24^ but this was not the case here. Similarly, Hu et al.^47^ showed that increasing diversity can reduce stability, making communities more vulnerable to environmental disturbances.

Interestingly, beta dispersion analysis indicated that there was no significant variation between the reactors in inter-sample variability when only abundance was considered, i.e. bray-curtis (Fig. 2). However, when phylogeny (unifrac) was used to assess beta dispersion (Fig. 2), significant (*p* < 0.05) differences were found between R1 and R3, indicating that the unstable conditions in R3 led to more phylogenetic dispersion in temporal samples in R3 over the course of the trial. The indication that phylogenetic composition (unifrac distance metric) was more variable between sampling timepoints in R3 is interesting. As the Unifrac distance metric does not consider abundance, it is a particularly useful metric for excluding the effect of the rare biosphere in diversity analysis^48^. The greater phylogenetic dispersion between sampling timepoints in R3, therefore not a result of fluctuations in rare taxa. However, rare taxa have been repeatedly suggested to play a role in resistance to environmental disturbances^49,50^ and functional redundancy. Therefore, the composition of the rare biosphere was analysed in further detail (Fig. 5).

Community assembly was largely stochastic across all reactors throughout the trial, which is contrary to many other studies which find that community assembly is largely deterministic in wastewater treatment systems and anaerobic digesters^51^. This indicates that the smaller disturbances applied to R2 and R3 throughout the trial did not have an effect on community assembly. However, a significant switch from stochastic to deterministic processes in R3 after the OLR shock occurred, which is similar to a previous study where recovery after an ammonia shock was governed by deterministic processes^52^.

In terms of taxonomic composition of the communities in R3, a large proportion of the temporally stable community was made up of the Spirochaetaceae family (Midas_g_14041) (Fig. 3). Since this analysis included all timepoints (including after the shock load), only taxa which were unaffected by the shock were thus identified. In fact the relative abundance of this family increased in the final sample, after the shock was applied, indicating that they have a degree of resistance. Spirochaetes are thought to have a fermentative or syntrophic acetate-oxidising functions in AD^53^. Methanolinea sp were the second and third most abundant taxa in the stable community of R1 and R2 respectively, but were absent from the stable community of R3. Methanolinea sp. were previously identified as being acid tolerant^54^ and therefore may have had a role in maintaining methane production in R1 and R2 after the influx of excess organic matter, and resultant acidogenesis (Supplementary Fig. 1).

In order to determine what may have led to the reduced resistance observed in R3, as a result of environmental instability, the functional and ecological properties of the community at T8, right before the applied shock were examined. Over the course of the trial, several MAGs increased in relative abundance in all three reactors, especially bin 57 of the genus *Tidjanibacter*, which was also the 4th most abundant bin overall. *Tidjanibacter* species are members of the *Rikenellaceae* family and were first isolated from the human colon^55^, although there have been suggestions that this species should be classified within the genus *Alistipes*^56^. *Tijanibacter* have also been found in the digestive systems of chickens^57^ and mice^58^ but to the best of the authors knowledge have not been identified in anaerobic digesters. Three members of the Patescibacteria phylum (bins 69, 198 and 348), also increased in abundance in all three reactors, but particularly in R1 and R2, especially bin 348, which was classified to order level as Moranbacteriales. The Patescibacteria are an uncultivated superphylum, which seem to be ubiquitous in many environments, but very little is known about their potential functions, other than that they have a small genome size and are thought to often be symbiotic with other microorganisms^59–61^, including methanogens of the *Methanothrix* genus^62^.

In terms of functional change in the recovered MAGs, the most striking distinction between the reactors was in TCRS (Fig. 8), where R3T8 clustered separately from the other samples (Fig. 8). TCRS are the molecular mechanisms which allow microbes to sense and respond to environmental stimuli by (de)activating the transcription of associated genes^63^. Two component systems typically consist of a membrane bound histidine kinase protein which senses environmental stimuli and a response regulator protein, which mediates differential gene expression^64^. Environmental stimuli could include the presence or absences of toxins and nutrients or physical characteristics such as redox state, pH and osmotic pressure^65^. Therefore it stands to reason that a microbial community which has had a more variable environment (i.e. R3) should have more TCSs. Indeed, 82 TCRS were more abundant in R3T8 than in R1T8 (Fig. 8). Several of the TCRSs which were more abundant in R3T8 have previously been associated with biofilm formation e.g. LuxQN/CqsS−LuxU−LuxO^66^, VicK−VicR^67^ and CiaH−CiaR^68^. This indicates that the unstable environment in R3 may have led to increased biofilm forming capacity. Indeed, the biomass dynamics in R3 differed from R1 or R2^23^ by producing much more biomass over the course of the trial (Supplementary Fig. 2).

TCRS which were more abundant in R1T8 included ArcB−ArcA (anoxic redox control) and HupT−HupR (hydrogenase synthesis regulation). The Arc two component system is utilised by facultative aerobes to detect and react to changes in respiratory growth conditions^69^ and regulates transcription of genes related to anaerobic growth in E.coli^70^. HupT-HupR TCRS has been mostly studied in *Rhodobacter* species, where it functions in conjunction with HupUV sensor protein in detecting and responding to H_2_ concentrations by producing hydrogenases, enabling a switch to autotrophic growth^71,72^. Therefore, R1 may have been more suited to dealing with changes in H_2_ concentrations and switching to hydrogenotrophic metabolism.

The contribution of these variations in TCRS to the failure of R3 is unclear and in fact their abundance may have had no impact at all on resistance (or the lack of). However, their predominance is indicative of the selection pressures of the applied disturbances, which has implications for further studies. Molecular diagnostics^73,74^ and modelling approaches^75,76^ have long been suggested to be the next step in process monitoring of anaerobic digestion, but no suitable target genes have so far been identified. The increase in the presence of TCRS in MAGs recovered from R3 indicates that they may be suitable for this purpose, particularly if detected at the transcriptomic level rather than at the genomic level. This would require further, similar studies where expression can be strongly linked to a given environmental disturbance. Once that is achieved, transcriptional screening could be carried out to assess community stress responses prior to process failure.

The absence of any conditionally rare taxa in R3 is surprising as environmental disturbances have previously been shown to promote temporal fluctuations in the rare biosphere^17,49^ and lead to the development of conditionally rare taxa^77^. In addition, our analysis indicated that persistently rare taxa made up a lower proportion of the total community in R3. Therefore, it may be the case that although these rare taxa did not increase enough in abundance to meet the threshold of conditionally rare taxa, they did increase to some extent, thus resulting in a lower proportion of persistently rare taxa. This also explains the observation that the number of other rare taxa were the highest in R3 (Fig. 5).

The taxa which shifted from persistently rare to other rare taxa may have been specialised to deal with a given disturbance (e.g. ammonia), but due to the short nature of the applied disturbances did not establish enough to become conditionally rare. Indeed, the unstable operation of R3 did lead to the proliferation of a microbial community with a higher proportion of specialist taxa than the control reactor (R1) (Fig. 6). The prevalence of specialist microbes in R3 was likely caused by the variety of applied disturbances, which led to the development of a larger number of narrow niches and less overlap (Supplementary Fig. 6) throughout the reactor operation. R2 had a similar number of specialist and generalist taxa to R1, indicating that the organic load increases in R2 did not lead to a proliferation of specialist microbes.

Generalist taxa have a wider niche breadth, and are able to survive under a wider set of conditions than specialists^78^. In contrast, specialists are more discriminant in the environments in which they thrive^41^. The relative proportion of generalists and specialists in a microbial community is thought to influence its stability in the face of a disturbance^79^. It was previously hypothesised^79^ that communities with a high proportion of specialist taxa would be more susceptible to functional breakdown in the face of a disturbance as the population performing the dominant function has a narrower niche breadth and will not be able to resist the change in conditions brought on by a disturbance. The present study provides evidence for this hypothesis in that the more specialist community of R3 failed in response to a disturbance, whereas the generalist communities of R1 and R2 were able to resist the applied shock.

The observation that different microbial life strategies evolved in the respective reactors and subsequent divergent responses of each reactor to a large disturbance indicates that a community made up of generalist microbes is more resistant to large disturbances. The proliferation of generalists was enhanced in R1 due to the stable environment. This is unexpected as generalist species are often thought to gain an advantage from environmental heterogeneity^13^. However, the distinct nature of each disturbance applied to R3 (i.e. pH, NH_3_, temperature and sulphate) may have led to the establishment of microbes which were specialised to deal with them.

Much more taxa overlap was observed in R1 and R2 (Supplementary Figs. 4 and 5) indicating that more interspecies interactions may have developed, potentially as a result of more consistent environmental conditions. Indeed a recent meta-analysis including pan-genomic data indicated that genomes of generalists are enriched in functions involved with species-species interactions^80^. The extent of interspecies interactions in a microbial community can impact its resistance to a disturbance as key interdependencies, e.g. sharing of public goods^81^ are buffered from sudden taxa die-off and ecosystem function is less reliant on a single keystone species, which may be inhibited by a disturbance^82,83^. It has also been suggested that lower numbers of interspecies interactions reduce ecosystem stability^84^ and increased interspecies interactions have even been shown to increase robustness in methanogenic communities^85^. Therefore, despite lower diversity, the generalist lifestyle which prevailed in the communities of R1 and R2 potentially increased their resistance to a major organic carbon influx.

A recent theory in microbial community ecology is that of ecological memory^86,87^. This area of research has been explored in plant ecology^88^ and is now being assessed in microbial communities, particularly soils^89^. The concept has been defined as ‘the capacity of past states or experiences to influence present or future responses of the community’^90^. In the context of this study, it could be considered that the range of disturbances applied to R2 and R3 led to the development of an ecological memory, which ultimately impacted future performance. However, similarly to much of the research on diversity-stability dynamics mentioned previously, it is largely considered that ecological memory of prior disturbances impart some degree of future resistance^89,91,92^, which was not the case for in the present study. This is an area of great relevance to environmental biotechnologies and should be explored further, to determine the effects of prior disturbances on future performance.

In conclusion, the variety of environmental disturbances applied to R3, led to a more variable microbial community, with higher diversity than R2, which received repeated OLR disturbances, or R1, which was maintained stable. The resultant community which evolved in R3 was more susceptible to deterministic influences on and less resistant to the shock load, despite having more abundant TCRS. This may have been due to the development of different niche spaces which were occupied by specialist taxa, whereas the stable environment in R1 led to proliferation of generalist taxa. Because specialist taxa were more abundant in R3, less overlap between respective taxa was detected, indicating that there were fewer interspecies interactions, which may also have made the system less robust to changing conditions. Overall these results indicate that environmental instability promoted the development of a microbial community which is less resistant to shocks, despite having higher diversity and a wider variety of TCRSs.

## Supplementary information

Supplemental Material

Supplemental File 1 metadata_amlicons

Supplemental File 2 metadata_metagenomes

Supplementary File 3 KEGG Sub Modules

Supplemental File 4 final_bin_statistics

## Data Availability

Raw sequencing data can be found in the National Center for Biotechnology Information (NCBI) Sequence Read Archive (SRA) under the BioProject accession number PRJNA1030189.

## Code availability

Some of the code used to generate the results are part of microbiomeSeq (https://github.com/umerijaz/microbiomeSeq), whilst other codes are available at http://userweb.eng.gla.ac.uk/umer.ijaz#bioinformatics. Furthermore, the data tables and code (adapted from above, and specific to this study) used in the analysis of amplicons and metagenomes is available on Zenodo (10.5281/zenodo.14845121).
