# Supplementary material for: Environmental instability reduces shock resistance by enriching specialist taxa with distinct two component regulatory systems
Source: NPJ Biofilms Microbiomes. 2025 Mar 31;11:54. doi: 10.1038/s41522-025-00679-w (PMC11958701; doi:10.1038/s41522-025-00679-w)
Supplement: Supplementary file 1 — Supplemental Material [file 41522_2025_679_MOESM1_ESM.pdf]

Environmental instability reduces shock resistance by enriching specialist taxa with distinct two component regulatory systems.

Simon Mills[1*], Umer Zeeshan Ijaz[1,2,3] Piet N.L. Lens[1]

[1]School of Chemical and Biological Sciences, National *University of Ireland, Galway, University Road, Galway, H91 TK33, Ireland*

[2]*Water & Environment Research Group, University of Glasgow, Mazumdar-Shaw Advanced Research Centre, Glasgow G11 6EW, United Kingdom*

[3]*Department of Molecular and Clinical Cancer Medicine, University of Liverpool, Liverpool L69 7BE, United Kingdom*

***Corresponding author**: Simon Mills, Ryan Institute, School of Biological and Chemical Sciences, University of Galway Simon Mills (simon.mills@universityofgalway.ie)

Submitted to: npj Biofilms and Microbiomes

1 Supplementary Methods

1.1 Local contribution to beta diversity

Local Contribution to Beta Diversity (LCBD) analysis [1] was conducted using LCBD.comp() from the adespatial package in R [2] with three different measures to assess beta diversity: (i) the Bray-Curtis distance (abundances), (ii) the unweighted unifrac distance (phylogeny), both estimated using the phyloseq package [3]. Additionally, (iii) Hierarchical Meta-Storms (HMS) [4] was also applied. HMS is a functional beta diversity distance which takes the observed KEGG Orthologs (KOs) (obtained via picrust2 in this case), and then calculates the functional beta diversity distance in a hierarchical fashion propagating the KOs abundances upward to the pathways in a multi-level pathway hierarchy to give a weighted dissimilarity measure. LCBD provides the sample-wise local contributions of individual samples to the total beta diversity. Thus, identifying how different the community of a single sample is from the mean beta diversity of all samples.

1.2 Beta Dispersion

We used Vegan's [5] betadisper() function to understand multivariate homogeneity of groups dispersion (variances) between multiple conditions, in which the distances between group centroids are handled by reducing the original distances (BrayCurtis, Unweighted Unifrac, HMS) to principal coordinates and then performing ANOVA on them. We used Vegan's adonis() for analysis of variance among sources of variation i.e. the Reactor.

1.3 Microbial Community Stability (EQO)

We used Ensemble Quotient Optimisation (EQO) [6] to find a stable and variable subset of taxa associated with each reactor. In this method a relative abundance table, called community matrix $\boldsymbol{M}$ (*P* OTUs over *n* samples), was used to obtain a vector $\boldsymbol{x} \in (0,1)^P$ where the *i^th^* position in the vector is either 0 or 1, i.e., a subset of species with values 1 belong to an ensemble which we are interested in recovering. This ensemble is identified with reference to a phenotype/predictor variable $\boldsymbol{y}$ by optimizing an *Ensemble Quotient* $EQ = \frac{\boldsymbol{x}^T\boldsymbol{Q}\boldsymbol{x}}{\boldsymbol{x}^T\boldsymbol{P}\boldsymbol{x}}$, through a genetic algorithm (an optimization algorithm), where $\boldsymbol{P}$ and $\boldsymbol{Q}$ are algebraic transformations of the community matrix that capture the

covariance between species, and the covariance between species and $\boldsymbol{y}$. The choice of $\boldsymbol{y}$ can be used in two cases: a) To recover an ensemble *that remains stable* for a set of samples, then $\boldsymbol{y}$ is considered uniform i.e., consisting of 1s, with $\boldsymbol{Q} = \boldsymbol{M}^T\mathbf{1}\mathbf{1}^T\boldsymbol{M}$, and $\boldsymbol{P} = \boldsymbol{M}^T\boldsymbol{M} - \frac{2}{n}\boldsymbol{M}^T\mathbf{1}\mathbf{1}^T\boldsymbol{M} + \frac{\mathbf{1}}{n^2}\boldsymbol{M}^T\mathbf{1}\mathbf{1}^T\mathbf{1}\mathbf{1}^T\boldsymbol{M}$ and b) To recover an ensemble whose cumulative abundance correlates with a continuous parameter y, then we optimize the algorithm with $\boldsymbol{Q} = \boldsymbol{M}_0^T\boldsymbol{y}_0\boldsymbol{y}_0^T\boldsymbol{M}_0$, $\boldsymbol{P} = \boldsymbol{M}_0^T\boldsymbol{M}_0$ ($\boldsymbol{M}_0$ is the centered community matrix $\boldsymbol{M}$ whose column means are zero with $\boldsymbol{y}_0$ also a centered version of $\boldsymbol{y}$).

Within the context of this study, we have used the case (a) to see which subset of microbes do not change over the whole time span of each reactor (quality of fit is returned as Coefficient of Variation CV), whilst case (b) was used to see which subset of microbes has a relationship with the sampling days (quality of fit is returned as a correlation coefficient between the continuous outcome and the cumulative abundance of the ensemble). To optimize the EQ to obtain $\boldsymbol{x}$, we followed the genetic algorithm optimization located at https://github.com/Xiaoyu2425/Ensemble-Quotient-Optimization. In the genetic algorithm, we have used the following parameterizations: a population size of 200 solutions, maximum of 400 generations, and maximum of 30 taxa to be returned as an ensemble.

1.4 Normalized Stochasticity Ratio and Nearest Taxa Index

The normalized stochasticity ratio (NST) was used to determine the influence of deterministic and stochastic community assembly processes with both Jaccard (incidence-based) and Ružička (abundance-based) metrics based on author recommendations [7]. Taxa-Richness constraints of proportional-proportional (P-P) and proportional-fixed (P-F) were applied for each metric. For this, the "tNST" function from the *NST* package in R was used [8]. When using abundance-based metric Ružička, null taxa abundances in each sample were calculated as random draw (1,000 times) of the observed number of individuals with probability proportional to regional relative abundances of null taxa in the treatments. The microbial community assembly is completely deterministic when NST is

0% and completely stochastic when NST is 100%. Detailed procedure for this analysis was described previously [9,10]

1.5 Nearest Taxa Index

Furthermore, we utilized the nearest taxa index (NTI) which, is a measure of mean pairwise phylogenetic distance. This method records original phylogenetic distances in a phylogenetic tree, and then generates 1000 randomizations of the phylogenetic tree (whilst keeping richness preserved) to calculate the mean nearest taxon distances on these distributions. Afterwards, the mean and standard deviation of these distances obtained from randomization procedure are used in a method called "statistical effect size" for comparison against the original distances to give NTI estimates. The values of NTI can be used to discern an underlying ecological mechanism. For a single community, NTI values > +2 suggest environmental filtering (local phylogenetic clustering), and values < -2 indicate competitive exclusion (local phylogenetic overdispersion) among species as the driver of community structure [11] . The detailed procedure for this analysis was described previously [10,12].

1.6 Rare Taxa

Methodology base on the work of [13] was to quantify abundant, conditionally rare, persistently rare and other rare ASVs. Abundant ASVs were defined as having an average relative abundance above 1% across all samples. Rare ASVs were defined as having an average relative abundance below 1% across all samples. Conditionally Rare ASVs exhibited a 100-fold change in their minimum and maximum relative abundance. Persistently Rare ASVs exhibited a maximum relative abundance exceeding 5-fold their minimum value. Other Rare ASVs exhibited a maximum relative abundance between 5 and 100 times greater than their minimum.

1.8 MicorNiche

To identify generalist (that should exist across all timepoints in a reactor) and specialist (that should exist at some timepoints in a reactor) microbes in each reactor, we have used R's MicroNiche package

[15]. Initially, genera were filtered out by using the limit of quantification (LOQ) approach as per the author's instruction. The LOQ approach filters taxa which are below a "decision boundary", calculated from the distribution of microbes with 95% certainty that these microbes will fall within a null distribution where the mean microbial abundance is zero. The null distribution standard deviation was calculated by fitting the lognormal rank distribution of the microbes with $S(R) = S_0 e^{-a^2 R^2}$ where log abundance of microbe S at rank R is dependent on coefficient a and rank R calculated as $a = \sqrt{\frac{\ln S_0}{S_m} / R^2}$ where S_m is the lowest taxon abundance of S. To calculate LOQ, we fit the above log normal model to data, and determined the LOQ as the overlap between the null hypothesis (i.e., a microbe's mean abundance is zero) and where the microbe falls within 1 standard deviation of the above model.

After filtering, niche breadth was calculated as Levins' $B_N = \frac{1}{R} \sum_{i=1} p_i^2$, where p_i is the proportional abundance of a genus in the i-th reactor, with total number of reactors (environments) being R (3 in this case). If B_N approaches 1 for a given genus, then it is considered as a "generalist", whilst if it approaches $1/R$, then it can be tagged as a "specialist". A null modelling approach was used to derive the p-value for Levins' B_N, where a random normal distribution of 999 possible niche breadths were produced for a genus. P values were then calculated based on whether a genus's B_N is greater or lower than the mean of the null model. As per the author's recommendation, after applying null modelling, the 5th Quantile and 95th Quantile were obtained to tag the genera as specialist if its B_N < 5th Quantile, and generalist, if its B_N > 95th Quantile. Those that fell in the inter-range were tagged as undecided.

In the second step, we then calculated the overlap of these undecided/specialist/generalists using Levins' Overlap formula $\mathrm{LO}_{i,j} = \frac{\sum_{i,j=1}(p_{ir})(p_{jr})}{\sum_{i=1}(p_{ir}^2)}$, where p_i is the proportional abundance of genus i in the r-th pen, and p_j is the abundance of genus j in the r-th pen, where i and j were selected after tagging an individual genus as undecided, specialist or generalist.

1.9 Heatmaps

For comparison of grouped features between treatment groups, sample-wise abundance tables were initially subjected into normalisation by total sum scaling (TSS) (number of reads for each MAG divided by total of reads per sample) and subsequently via centralised log ratio (CLR) method using the logratio.transfo function (log-ratio transformation) of the mixOmics package (Rohart et al., 2017). Subsequently, we used R's pheatmap to draw the heatmap of selective pathways along with metadata superimposed on top of the heatmap

2 Supplementary Results

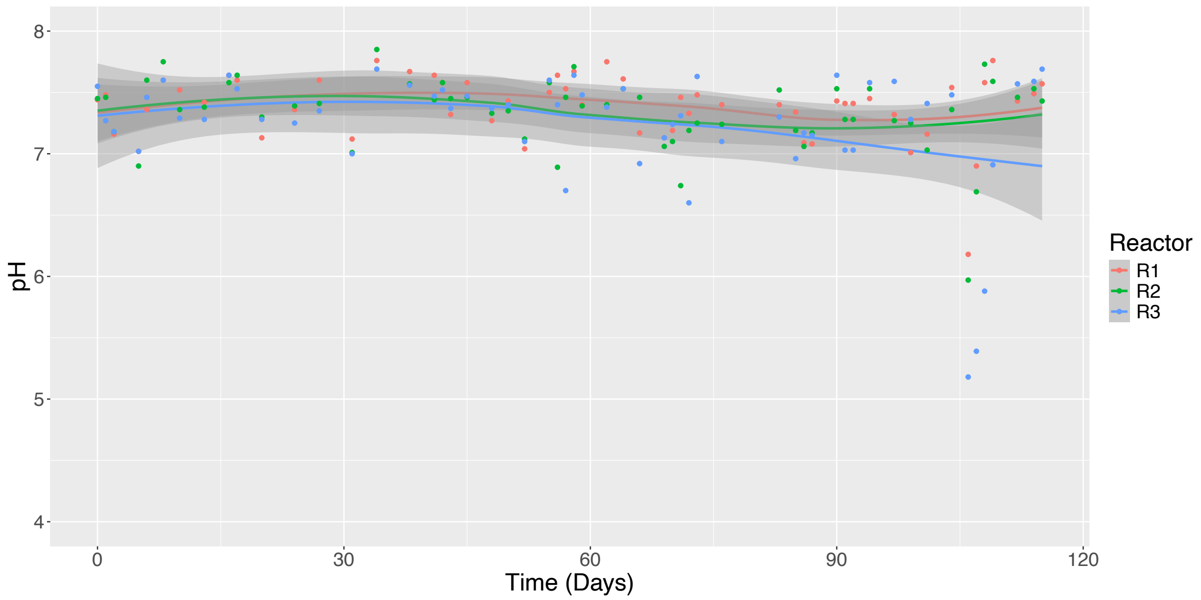

Supplementary Figure 1. Reactor pH. pH values including local regression lines for each reactor over the course of the trial.

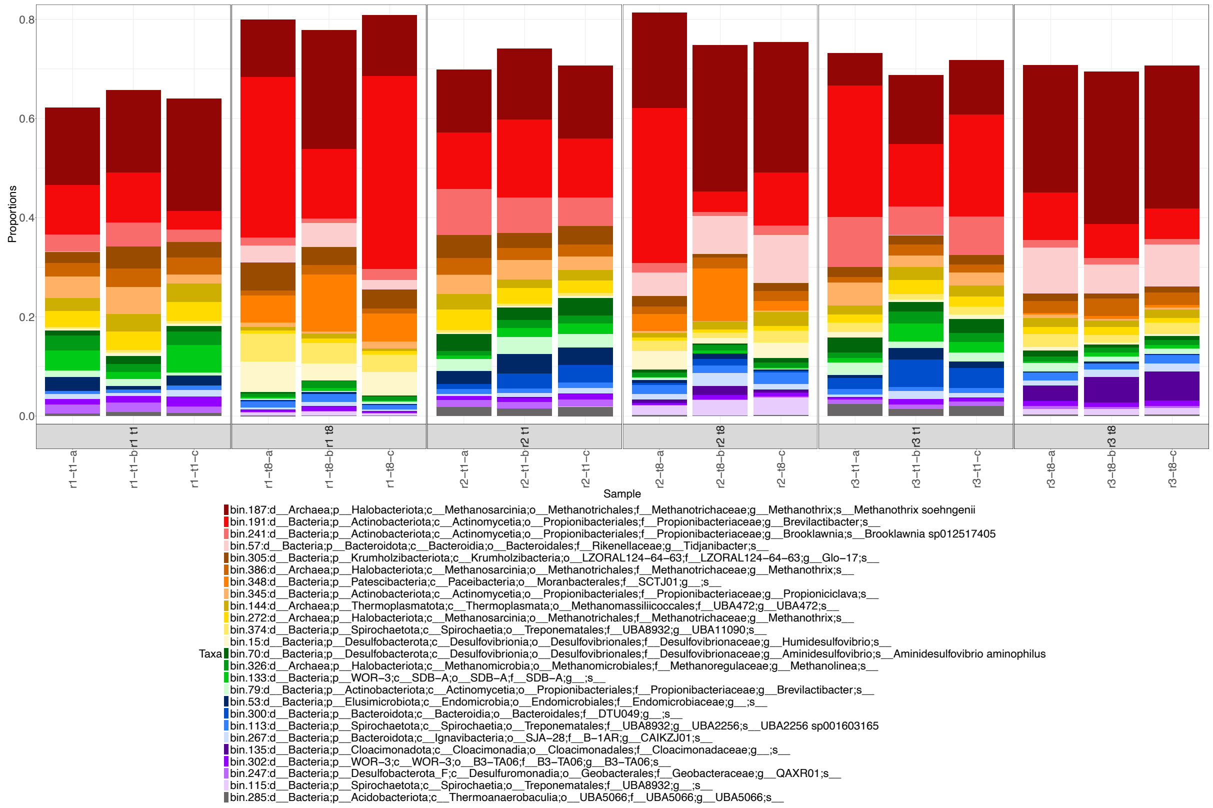

Supplementary Figure 2. Abundant MAGS. The 25 most abundant MAGs across all samples are presented in each sample.

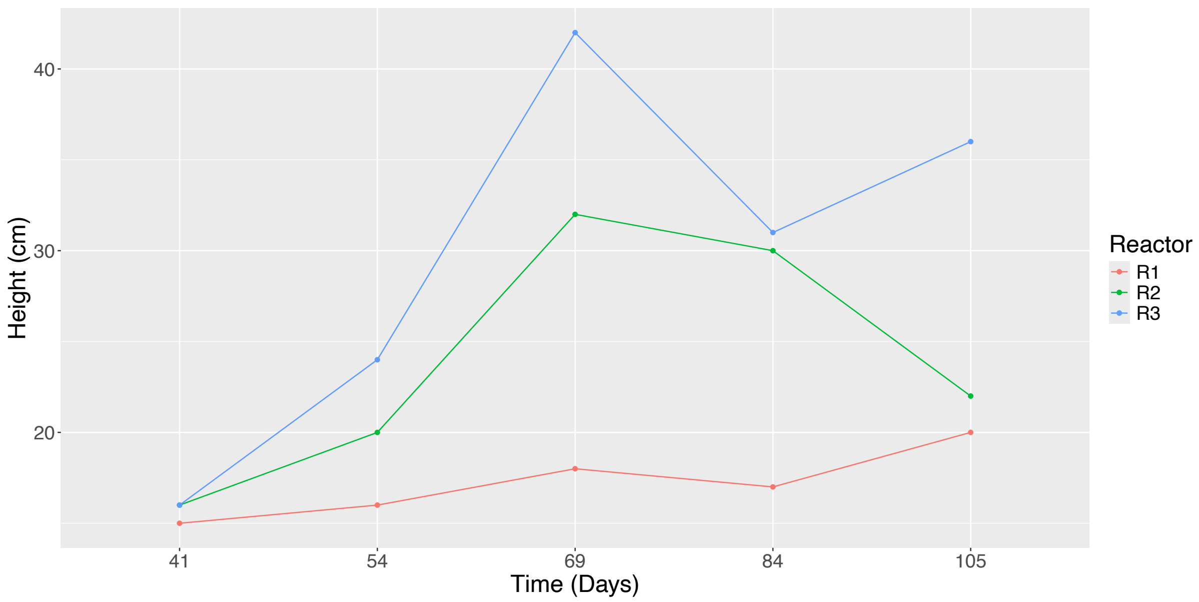

Supplementary Figure 3. Sludge Bed. Sludge bed height (cm) over the course of the trial in each reactor.

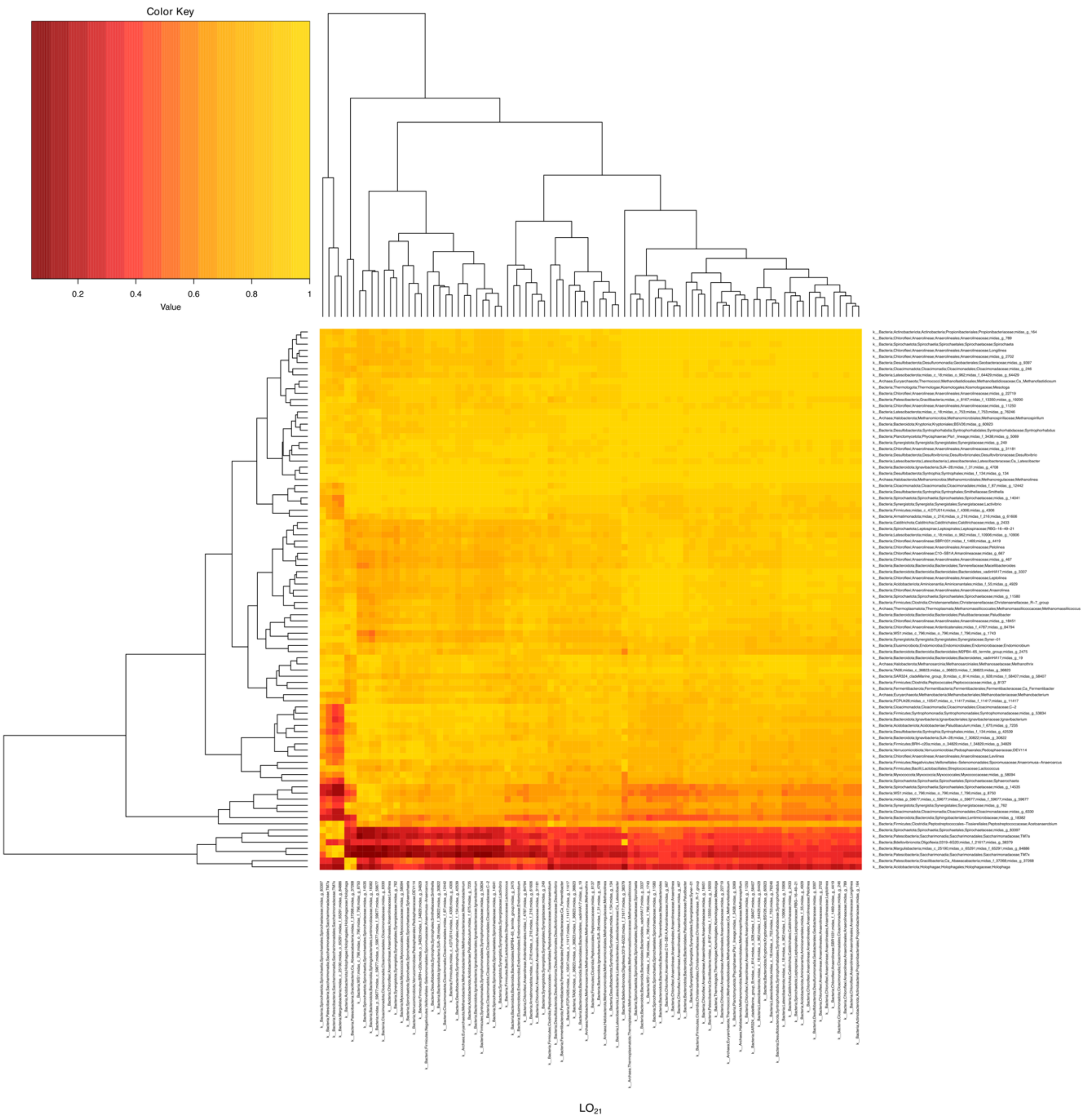

Supplementary Figure 4. Reactor 1 Taxa Overlap. The Proportional Levin's Overlap PLO_(i,j) between generalists and specialists which approaches 0 when pairs are inversely related to each other, and approaches 1 for genus pairs that are positively related to each other in R1.

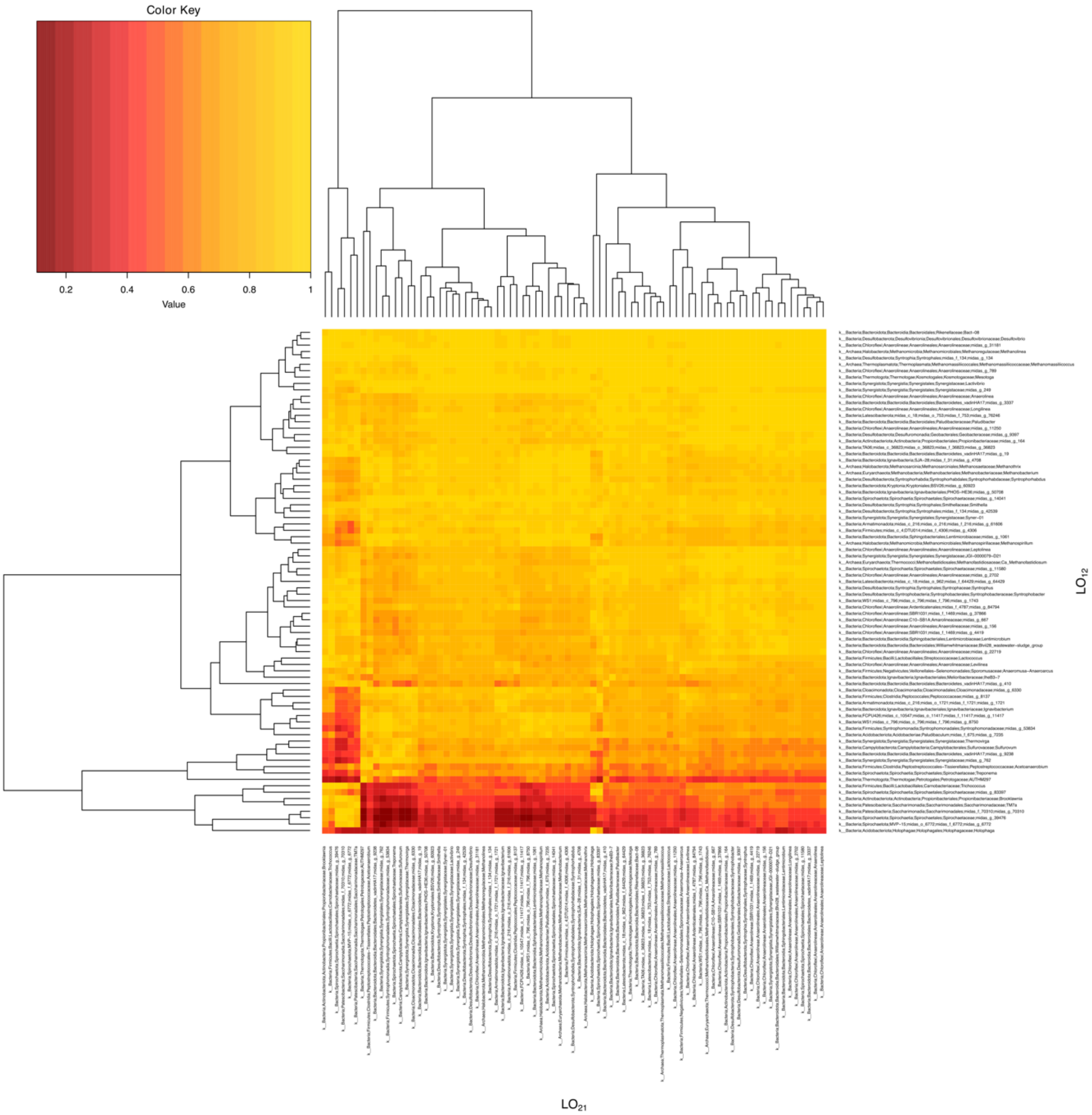

Supplementary Figure 5. Reactor 2 Taxa Overlap. The Proportional Levin's Overlap PLO_(i,j) between generalists and specialists which approaches 0 when pairs are inversely related to each other, and approaches 1 for genus pairs that are positively related to each other in R2.

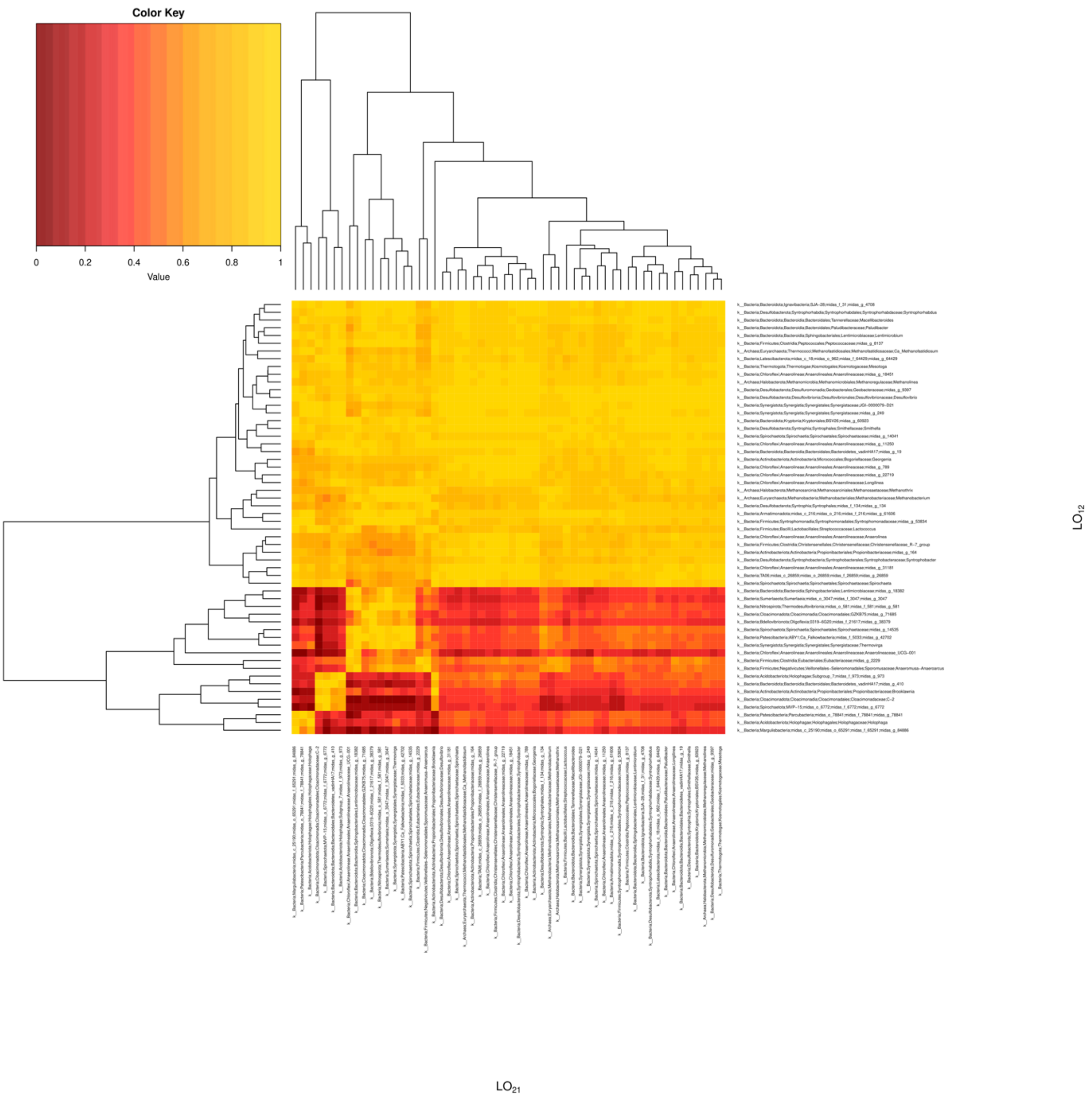

Supplementary Figure 6. Reactor 2 Taxa Overlap. The Proportional Levin's Overlap PLO_(i,j) between generalists and specialists which approaches 0 when pairs are inversely related to each other, and approaches 1 for genus pairs that are positively related to each other in R3.

Supplementary Table 1. Generalist and specialist taxa in R1, R2 and R3

Node	Taxa	R1 Classification	R2 Classification	R3 Classification
1	Bacteria;Firmicutes;Clostridia;Eubacteriales;Anaerofustaceae;Anaerofustis	Generalist		
2	Bacteria;Fermentibacterota;Fermentibacteria;Fermentibacterales;Fermentibacteraceae;Ca_Fermentibacter	Generalist		
3	Bacteria;Planctomycetota;Phycisphaerae;Pla1_lineage;midas_f_3438;midas_g_5069	Generalist		
4	Bacteria;midas_p_59677;midas_c_59677;midas_o_59677;midas_f_59677;midas_g_59677	Generalist		
5	Bacteria;Spirochaetota;Spirochaetia;Spirochaetales;Spirochaetaceae;Sphaerochaeta	Generalist		
6	Bacteria;Latescibacterota;Latescibacteria;Latescibacterales;Latescibacteraceae;Ca_Latescibacter	Generalist		
7	Bacteria;Spirochaetota;Leptospirae;Leptospirales;Leptospiraceae;RBG-16-49-21	Generalist		
8	Bacteria;Acidobacteriota;Aminicenantia;Aminicenantales;midas_f_55;midas_g_4929	Generalist		
9	Bacteria;Bacteroidota;Ignavibacteria;SJA-28;midas_f_30822;midas_g_30822	Generalist		
10	Bacteria;Chloroflexi;Anaerolineae;Anaerolineales;Anaerolineaceae;Pelolinea	Generalist		
11	Bacteria;Calditrichota;Calditrichia;Calditrichales;Calditrichaceae;midas_g_2433	Generalist		
12	Bacteria;Margulisbacteria;midas_c_25190;midas_o_65291;midas_f_65291;midas_g_84886	Specialist		Specialist
13	Bacteria;Myxococcota;Myxococcia;Myxococcales;Myxococcaceae;midas_g_58094	Generalist		
14	Bacteria;Bacteroidota;Bacteroidia;Sphingobacteriales;Lentimicrobiaceae;midas_g_18382	Generalist		Specialist
15	Bacteria;Acidobacteriota;Acidobacteriae;Paludibaculum;midas_f_675;midas_g_7235	Generalist	Generalist	
16	Bacteria;WS1;midas_c_796;midas_o_796;midas_f_796;midas_g_8750	Generalist	Generalist	
17	Bacteria;WS1;midas_c_796;midas_o_796;midas_f_796;midas_g_1743	Generalist	Generalist	
18	Bacteria;Bacteroidota;Bacteroidia;Bacteroidales;M2PB4-65_termite_group;midas_g_2475	Generalist		
19	Bacteria;Bacteroidota;Ignavibacteria;Ignavibacteriales;Ignavibacteriaceae;Ignavibacterium	Generalist	Generalist	
20	Bacteria;Synergistota;Synergistia;Synergistales;Synergistaceae;Syner-01	Generalist	Generalist	
21	Bacteria;Firmicutes;Clostridia;Peptostreptococcales-Tissierellales;Peptostreptococcaceae;Acetoanaerobium	Generalist	Generalist	
22	Bacteria;Armatimonadota;midas_c_216;midas_o_216;midas_f_216;midas_g_61606	Generalist	Generalist	Generalist
23	Bacteria;Spirochaetota;Spirochaetia;Spirochaetales;Spirochaetaceae;Spirochaeta	Generalist		Generalist
24	Bacteria;SAR324_cladeMarine_group_B;midas_c_814;midas_o_928;midas_f_58407;midas_g_58407	Generalist		
25	Bacteria;Latescibacterota;midas_c_18;midas_o_962;midas_f_10906;midas_g_10906	Generalist		
26	Bacteria;Bdellovibrionota;Oligoflexia;0319-6G20;midas_f_21617;midas_g_38379	Specialist		Specialist
27	Bacteria;Chloroflexi;Anaerolineae;SBR1031;midas_f_1469;midas_g_4419	Generalist	Generalist	

28	Bacteria;Bacteroidota;Kryptonia;Kryptoniales;BSV26;midas_g_60923	Generalist	Generalist	Generalist
29	Bacteria;FCPU426;midas_c_10547;midas_o_11417;midas_f_11417;midas_g_11417	Generalist	Generalist	
30	Bacteria;Chloroflexi;Anaerolineae;Anaerolineales;Anaerolineaceae;midas_g_18451	Generalist		Generalist
31	Bacteria;Patescibacteria;Saccharimonadia;Saccharimonadales;Saccharimonadaceae;TM7x		Specialist	
32	Bacteria;Firmicutes;Clostridia;Peptococcales;Peptococcaceae;midas_g_8137	Generalist	Generalist	Generalist
33	Bacteria;Firmicutes;midas_c_4;DTU014;midas_f_4306;midas_g_4306	Generalist	Generalist	
34	Bacteria;Desulfobacterota;Syntrophia;Syntrophales;midas_f_134;midas_g_42539	Generalist	Generalist	
35	Bacteria;Cloacimonadota;Cloacimonadia;Cloacimonadales;midas_f_87;midas_g_12442	Generalist		
36	Bacteria;Chloroflexi;Anaerolineae;Ardenticatenales;midas_f_4787;midas_g_84794	Generalist	Generalist	
37	Bacteria;Verrucomicrobiota;Verrucomicrobiae;Pedosphaerales;Pedosphaeraceae;DEV114	Generalist		
38	Bacteria;Cloacimonadota;Cloacimonadia;Cloacimonadales;Cloacimonadaceae;C-2	Generalist		Specialist
39	Bacteria;Latescibacterota;midas_c_18;midas_o_962;midas_f_64429;midas_g_64429	Generalist	Generalist	Generalist
40	Bacteria;Firmicutes;Bacilli;Lactobacillales;Streptococcaceae;Lactococcus	Generalist	Generalist	Generalist
41	Bacteria;Firmicutes;BRH-c20a;midas_o_34829;midas_f_34829;midas_g_34829	Generalist		
42	Bacteria;Synergistota;Synergistia;Synergistales;Synergistaceae;midas_g_762	Generalist	Generalist	
43	Bacteria;Chloroflexi;Anaerolineae;Anaerolineales;Anaerolineaceae;midas_g_467	Generalist		
44	Bacteria;Spirochaetota;Spirochaetia;Spirochaetales;Spirochaetaceae;midas_g_83397	Specialist	Specialist	
45	Bacteria;Patescibacteria;Gracilibacteria;Ca_Abawacabacteria;midas_f_37268;midas_g_37268		Specialist	
46	Bacteria;Chloroflexi;Anaerolineae;Anaerolineales;Anaerolineaceae;Levilinea	Generalist	Generalist	
47	Bacteria;Patescibacteria;Saccharimonadia;Saccharimonadales;Saccharimonadaceae;TM7a	Specialist	Specialist	
48	Bacteria;Chloroflexi;Anaerolineae;Anaerolineales;Anaerolineaceae;midas_g_2702	Generalist	Generalist	
49	Archaea;Halobacterota;Methanomicrobia;Methanomicrobiales;Methanospirillaceae;Methanospirillum	Generalist	Generalist	
50	Bacteria;Spirochaetota;Spirochaetia;Spirochaetales;Spirochaetaceae;midas_g_11580	Generalist	Generalist	
51	Bacteria;Firmicutes;Negativicutes;Veillonellales-Selenomonadales;Sporomusaceae;Anaeromusa-Anaeroarcus	Generalist	Generalist	Generalist
52	Bacteria;Acidobacteriota;Holophagae;Holophagales;Holophagaceae;Holophaga	Specialist	Specialist	Specialist
53	Bacteria;TA06;midas_c_36823;midas_o_36823;midas_f_36823;midas_g_36823	Generalist	Generalist	
54	Bacteria;Desulfobacterota;Syntrophia;Syntrophales;midas_f_134;midas_g_134	Generalist	Generalist	Generalist
55	Archaea;Euryarchaeota;Thermococci;Methanofastidiosales;Methanofastidiosaceae;Ca_Methanofastidiosum	Generalist	Generalist	Generalist
56	Bacteria;Cloacimonadota;Cloacimonadia;Cloacimonadales;Cloacimonadaceae;midas_g_246	Generalist		
57	Bacteria;Bacteroidota;Bacteroidia;Bacteroidales;Bacteroidetes_vadinHA17;midas_g_19	Generalist	Generalist	Generalist
58	Bacteria;Bacteroidota;Bacteroidia;Bacteroidales;Bacteroidetes_vadinHA17;midas_g_3337	Generalist	Generalist	
59	Bacteria;Chloroflexi;Anaerolineae;Anaerolineales;Anaerolineaceae;midas_g_22719	Generalist	Generalist	Generalist

60	Bacteria;Thermotogota;Thermotogae;Kosmotogales;Kosmotogaceae;Mesotoga	Generalist	Generalist	Generalist
61	Bacteria;Bacteroidota;Bacteroidia;Bacteroidales;Tannerellaceae;Macellibacteroides	Generalist		Generalist
62	Bacteria;Chloroflexi;Anaerolineae;Anaerolineales;Anaerolineaceae;Leptolinea	Generalist	Generalist	
63	Bacteria;Spirochaetota;Spirochaetia;Spirochaetales;Spirochaetaceae;midas_g_14535	Generalist		Specialist
64	Bacteria;Desulfobacterota;Desulfovibrionia;Desulfovibrionales;Desulfovibrionaceae;Desulfovibrio	Generalist	Generalist	Generalist
65	Bacteria;Bacteroidota;Ignavibacteria;SJA-28;midas_f_31;midas_g_4708	Generalist	Generalist	Generalist
66	Bacteria;Latescibacterota;midas_c_18;midas_o_753;midas_f_753;midas_g_76246	Generalist	Generalist	
67	Bacteria;Bacteroidota;Bacteroidia;Bacteroidales;Paludibacteraceae;Paludibacter	Generalist	Generalist	Generalist
68	Bacteria;Elusimicrobiota;Endomicrobia;Endomicrobiales;Endomicrobiaceae;Endomicrobium	Generalist		
69	Archaea;Thermoplasmatota;Thermoplasmata;Methanomassiliicoccales;Methanomassiliicoccaceae;Methanomassiliicoccus	Generalist	Generalist	
70	Archaea;Euryarchaeota;Methanobacteria;Methanobacteriales;Methanobacteriaceae;Methanobacterium	Generalist	Generalist	Generalist
71	Bacteria;Chloroflexi;Anaerolineae;C10-SB1A;Amarolineaceae;midas_g_667	Generalist	Generalist	
72	Bacteria;Synergistota;Synergistia;Synergistales;Synergistaceae;Lactivibrio	Generalist	Generalist	
73	Bacteria;Firmicutes;Syntrophomonadia;Syntrophomonadales;Syntrophomonadaceae;midas_g_53834	Generalist	Generalist	Generalist
74	Archaea;Halobacterota;Methanomicrobia;Methanomicrobiales;Methanoregulaceae;Methanolinea	Generalist	Generalist	Generalist
75	Bacteria;Desulfobacterota;Syntrophia;Syntrophales;Smithellaceae;Smithella	Generalist	Generalist	Generalist
76	Bacteria;Chloroflexi;Anaerolineae;Anaerolineales;Anaerolineaceae;midas_g_789	Generalist	Generalist	Generalist
77	Bacteria;Patescibacteria;Gracilibacteria;midas_o_8167;midas_f_13350;midas_g_19200	Generalist		
78	Bacteria;Chloroflexi;Anaerolineae;Anaerolineales;Anaerolineaceae;Anaerolinea	Generalist	Generalist	Generalist
79	Bacteria;Chloroflexi;Anaerolineae;Anaerolineales;Anaerolineaceae;midas_g_11250	Generalist	Generalist	Generalist
80	Bacteria;Chloroflexi;Anaerolineae;Anaerolineales;Anaerolineaceae;Longilinea	Generalist	Generalist	Generalist
81	Bacteria;Firmicutes;Clostridia;Christensenellales;Christensenellaceae;Christensenellaceae_R-7_group	Generalist		Generalist
82	Bacteria;Desulfobacterota;Desulfuromonadia;Geobacterales;Geobacteraceae;midas_g_9397	Generalist	Generalist	Generalist
83	Bacteria;Spirochaetota;Spirochaetia;Spirochaetales;Spirochaetaceae;midas_g_14041	Generalist	Generalist	Generalist
84	Bacteria;Chloroflexi;Anaerolineae;Anaerolineales;Anaerolineaceae;midas_g_31181	Generalist	Generalist	Generalist
85	Bacteria;Cloacimonadota;Cloacimonadia;Cloacimonadales;Cloacimonadaceae;midas_g_6330	Generalist	Generalist	
86	Bacteria;Actinobacteriota;Actinobacteria;Propionibacteriales;Propionibacteriaceae;midas_g_164	Generalist	Generalist	Generalist
87	Bacteria;Desulfobacterota;Syntrophorhabdia;Syntrophorhabdales;Syntrophorhabdaceae;Syntrophorhabdus	Generalist	Generalist	Generalist
88	Bacteria;Synergistota;Synergistia;Synergistales;Synergistaceae;midas_g_249	Generalist	Generalist	Generalist
89	Archaea;Halobacterota;Methanosarcinia;Methanosarciniales;Methanosaetaceae;Methanothrix	Generalist	Generalist	Generalist
90	Bacteria;Myxococcota;Polyangia;Polyangiales;Phaselicystidaceae;Phaselicystis		Specialist	
91	Bacteria;Campylobacterota;Campylobacteria;Campylobacterales;Sulfurovaceae;Sulfurovum		Generalist	

92	Bacteria;Spirochaetota;Spirochaetia;Spirochaetales;Spirochaetaceae;Treponema		Generalist	
93	Bacteria;Spirochaetota;Spirochaetia;Spirochaetales;Spirochaetaceae;midas_g_39476		Specialist	
94	Bacteria;Patescibacteria;Saccharimonadia;Saccharimonadales;midas_f_70310;midas_g_70310		Specialist	
95	Bacteria;Bacteroidota;Bacteroidia;Bacteroidales;Bacteroidetes_vadinHA17;midas_g_410		Generalist	Specialist
96	Bacteria;Chloroflexi;Anaerolineae;SBR1031;midas_f_1469;midas_g_37866		Generalist	
97	Bacteria;Spirochaetota;MVP-15;midas_o_6772;midas_f_6772;midas_g_6772		Specialist	Specialist
98	Bacteria;Thermotogota;Thermotogae;Petrotogales;Petrotogaceae;AUTHM297		Generalist	
99	Bacteria;Actinobacteriota;Actinobacteria;Propionibacteriales;Propionibacteriaceae;Brooklawnia		Specialist	Specialist
100	Bacteria;Chloroflexi;Anaerolineae;Anaerolineales;Anaerolineaceae;midas_g_156		Generalist	
101	Bacteria;Bacteroidota;Bacteroidia;Bacteroidales;Bacteroidetes_vadinHA17;midas_g_9238		Generalist	
102	Bacteria;Bacteroidota;Ignavibacteria;Ignavibacteriales;PHOS-HE36;midas_g_50708		Generalist	
103	Bacteria;Armatimonadota;midas_c_216;midas_o_1721;midas_f_1721;midas_g_1721		Generalist	
104	Bacteria;Firmicutes;Bacilli;Lactobacillales;Carnobacteriaceae;Trichococcus		Specialist	
105	Bacteria;Desulfobacterota;Syntrophia;Syntrophales;Syntrophaceae;Syntrophus		Generalist	
106	Bacteria;Synergistota;Synergistia;Synergistales;Synergistaceae;Thermovirga		Generalist	Specialist
107	Bacteria;Desulfobacterota;Syntrophobacteria;Syntrophobacterales;Syntrophobacteraceae;Syntrophobacter		Generalist	Generalist
108	Bacteria;Bacteroidota;Bacteroidia;Sphingobacteriales;Lentimicrobiaceae;midas_g_1061		Generalist	
109	Bacteria;Bacteroidota;Bacteroidia;Bacteroidales;Williamwhitmaniaceae;Blvii28_wastewater-sludge_group		Generalist	
110	Bacteria;Bacteroidota;Bacteroidia;Bacteroidales;Rikenellaceae;Bact-08		Generalist	
111	Bacteria;Bacteroidota;Ignavibacteria;Ignavibacteriales;Melioribacteraceae;IheB3-7		Generalist	
112	Bacteria;Bacteroidota;Bacteroidia;Sphingobacteriales;Lentimicrobiaceae;Lentimicrobium		Generalist	Generalist
113	Bacteria;Synergistota;Synergistia;Synergistales;Synergistaceae;JGI-0000079-D21		Generalist	Generalist
114	Bacteria;Spirochaetota;Spirochaetia;Spirochaetales;Spirochaetaceae;midas_g_7329			Specialist
115	Bacteria;Acidobacteriota;Holophagae;Subgroup_7;midas_f_973;midas_g_973			Specialist
116	Bacteria;Sumerlaeota;Sumerlaeia;midas_o_3047;midas_f_3047;midas_g_3047			Specialist
117	Bacteria;Patescibacteria;ABY1;Ca_Falkowbacteria;midas_f_5033;midas_g_42702			Specialist
118	Bacteria;Patescibacteria;Parcubacteria;midas_o_78841;midas_f_78841;midas_g_78841			Specialist
119	Bacteria;Actinobacteriota;Actinobacteria;Micrococcales;Bogoriellaceae;Georgenia			Generalist
120	Bacteria;Chloroflexi;Anaerolineae;Anaerolineales;Anaerolineaceae;Anaerolineaceae_UCG-001			Specialist
121	Bacteria;Nitrospirota;Thermodesulfovibrionia;midas_o_581;midas_f_581;midas_g_581			Specialist
122	Bacteria;Cloacimonadota;Cloacimonadia;Cloacimonadales;GZKB75;midas_g_71685			Specialist
123	Bacteria;Firmicutes;Clostridia;Eubacteriales;Eubacteriaceae;midas_g_2229			Generalist

124	Bacteria;TA06;midas_c_26859;midas_o_26859;midas_f_26859;midas_g_26859			Generalist

Supplementary References

1. Legendre, P. & De Cáceres, M. Beta diversity as the variance of community data: dissimilarity coefficients and partitioning. *Ecol. Lett.* **16**, 951–963 (2013).

2. Dray, S. *et al.* adespatial: Multivariate multiscale spatial analysis. *R Packag. version 0.0* **3**, (2016).

3. McMurdie, P. J. & Holmes, S. phyloseq: An R Package for Reproducible Interactive Analysis and Graphics of Microbiome Census Data. *PLoS One* **8**, 1–11 (2013).

4. Zhang, Y., Jing, G., Chen, Y., Li, J. & Su, X. Hierarchical Meta-Storms enables comprehensive and rapid comparison of microbiome functional profiles on a large scale using hierarchical dissimilarity metrics and parallel computing. *Bioinforma. Adv.* **1**, vbab003 (2021).

5. Oksanen, J. *et al.* Vegan: community ecology package. R Package version 2.2-1. (2015).

6. Shan, X., Goyal, A., Gregor, R. & Cordero, O. X. Annotation-free discovery of functional groups in microbial communities. *Nat. Ecol. Evol.* **7**, 716–724 (2023).

7. Ning, D., Deng, Y., Tiedje, J. M. & Zhou, J. A general framework for quantitatively assessing ecological stochasticity. *Proc. Natl. Acad. Sci.* **116**, 16892–16898 (2019).

8. Ning, D. *et al.* A quantitative framework reveals ecological drivers of grassland microbial community assembly in response to warming. *Nat. Commun.* **11**, 4717 (2020).

9. Carboni, M. F. *et al.* Autotrophic denitrification of nitrate rich wastewater in fluidized bed reactors using pyrite and elemental sulfur as electron donors. *Environ. Technol. Innov.* **28**, 102878 (2022).

10. Nikolova, C., Ijaz, U. Z. & Gutierrez, T. Exploration of marine bacterioplankton community assembly mechanisms during chemical dispersant and surfactant-assisted oil biodegradation. *Ecol. Evol.* **11**, 13862–13874 (2021).

11. Stegen, J. C., Lin, X., Konopka, A. E. & Fredrickson, J. K. Stochastic and deterministic assembly processes in subsurface microbial communities. *ISME J.* **6**, 1653–1664 (2012).

12. Mills, S. *et al.* A Distinct, Flocculent, Acidogenic Microbial Community Accompanies Methanogenic Granules in Anaerobic Digesters. *Microbiol. Spectr.* **0**, e00784-21 (2021).

13. Yang, S., Winkel, M., Wagner, D. & Liebner, S. Community structure of rare methanogenic archaea: insight from a single functional group. *FEMS Microbiol. Ecol.* **93**, fix126 (2017).

14. Darcy, J. L., Amend, A. S., Swift, S. O. I., Sommers, P. S. & Lozupone, C. A. specificity: an R package for analysis of feature specificity to environmental and higher dimensional variables, applied to microbiome species data. *bioRxiv* 2021.11.06.467582 (2022). doi:10.1101/2021.11.06.467582

15. Finn, D. R. *et al.* MicroNiche: an R package for assessing microbial niche breadth and overlap from amplicon sequencing data. *FEMS Microbiol. Ecol.* **96**, (2020).
